# Automatic Childhood Pneumonia Diagnosis Based on Multi-Model Feature Fusion Using Chi-Square Feature Selection

**DOI:** 10.3390/jimaging12020081

**Published:** 2026-02-14

**Authors:** Amira Ouerhani, Tareq Hadidi, Hanene Sahli, Halima Mahjoubi

**Affiliations:** 1 Research Laboratory of Biophysics and Medical Technologies, Higher Institute of Medical Technologies of Tunis, University of Tunis El Manar, Tunis 1006, Tunisia; halima.mahjoubi@utm.tn; 2 Biomedical Technology Department, Applied Medical Sciences College, Prince Sattam Bin Abdulaziz University, Al-Kharj 11942, Saudi Arabia; t.hadidi@psau.edu.sa; 3 Laboratory of Signal Image and Energy Mastery, National Higher School of Engineers of Tunis, University of Tunis, Tunis 1008, Tunisia; sahli.hanenne@gmail.com

**Keywords:** pneumonia detection, feature selection, feature concatenation, ensemble model, Grad-CAM, Chi-square test

## Abstract

Pneumonia is one of the main reasons for child mortality, with chest radiography (CXR) being essential for its diagnosis. However, the low radiation exposure in pediatric analysis complicates the accurate detection of pneumonia, making traditional examination ineffective. Progress in medical imaging with convolutional neural networks (CNN) has considerably improved performance, gaining widespread recognition for its effectiveness. This paper proposes an accurate pneumonia detection method based on different deep CNN architectures that combine optimal feature fusion. Enhanced VGG-19, ResNet-50, and MobileNet-V2 are trained on the most widely used pneumonia dataset, applying appropriate transfer learning and fine-tuning strategies. To create an effective feature input, the Chi-Square technique removes inappropriate features from every enhanced CNN. The resulting subsets are subsequently fused horizontally, to generate more diverse and robust feature representation for binary classification. By combining 1000 best features from VGG-19 and MobileNet-V2 models, the suggested approach records the best accuracy (97.59%), Recall (98.33%), and F1-score (98.19%) on the test set based on the supervised support vector machines (SVM) classifier. The achieved results demonstrated that our approach provides a significant enhancement in performance compared to previous studies using various ensemble fusion techniques while ensuring computational efficiency. We project this fused-feature system to significantly aid timely detection of childhood pneumonia, especially within constrained healthcare systems.

## 1. Introduction

Pneumonia remains a major cause of morbidity and mortality worldwide, with approximately 450 million cases and 4 million deaths reported annually [1]. It commonly affects the lungs and results from infection by bacteria, viruses, or other harmful agents, leading to inflammation of the alveoli and surrounding lung parenchyma. This respiratory illness can be life-threatening, especially for children under 5 [2]. Hence, pneumonia continues to be a major contributor to childhood mortality, especially in developing and underdeveloped countries, where limited access to healthcare contributes to high mortality rates [3]. As a result, timely detection of pneumonia is essential to provide adequate therapy and enhance survival chances.

Thoracic Radiography also known as Chest imaging (CXR), remains the primary technique currently used for the rapid diagnosis of pneumonia. During, traditional examination, radiologists identify abnormalities such as opacities that indicate fluid accumulation in lung regions. However, manual procedures are labor-intensive and highly dependent on clinical settings and practitioner experience, leading to significant inter-observer variability, particularly in borderline cases [4]. Detecting childhood pneumonia is even more difficult due to the low radiation levels used in pediatric imaging and the challenge of capturing high-quality X-rays in proper posture, especially with very young patients who may not cooperate during imaging. These limitations highlight the need for automated computer-aided diagnostic (CAD) systems, which are crucial for assisting radiologists and clinicians in reaching more accurate diagnoses.

Recent advances in artificial intelligence (AI), particularly deep learning, have significantly improved automated medical image analysis. Convolutional neural networks (CNNs) have demonstrated strong performance in diagnosing various disorders, including pneumonia [5,6,7]. These architectures have the potential to identify prominent characteristics of a vast number of images, without requiring manual domain expertise like traditional machine learning (ML). These features, though not directly interpretable, effectively distinguish image patterns [5].

In medical imaging, the common practice is to adopt CNN architectures that reached top performance in the ImageNet Large Scale Visual Recognition Challenge (ILSVRC), such as ResNet, Inception, or VGG. The use of these models on smaller datasets effectively reduces the computational cost and effort of training and optimizing a model from scratch. Despite their success, individual CNN models often capture only a narrow set of feature representations, which may limit their ability to detect subtle and diverse pathological patterns [6,7]. In such cases, Deep ensemble model can be employed to enhance feature diversity by combining the strengths of diverse pre-trained architectures.

While common ensemble methods operating at the decision level (e.g., average probability, weighted probability, and majority voting) improve performance compared to individual CNNs, they often ignore valuable internal feature representations, limiting their effectiveness. For this reason, methods such as multi-model feature concatenation are proving more effective in improving detection accuracy and allowing models to generalize better [8]. However, processing the combined feature sets poses a major technical challenge due to high dimensionality, which can adversely affect overall performance because of redundant or non-informative features. Although feature selection techniques aim to mitigate this issue, existing studies rarely focus on selecting the most relevant feature subsets from each model within a deep ensemble framework.

In light of these gaps, this work proposes the Chi-square Selected Feature Fusion Ensemble (CSFFE), a DL ensemble model that selectively identifies and concatenates the most relevant features extracted from VGG-19, ResNet-50, and MobileNet-V2. By applying Chi-square-based feature selection prior to feature fusion, the proposed approach produces a compact and highly discriminative representation that enhances classification performance while reducing unnecessary complexity. Pneumonia diagnosis is formulated as a binary image-level classification task, assigning each chest X-ray image to either the pneumonia or normal class.

In this context, we investigate whether exploiting complementary feature representations learned by three well-established CNN models through feature-level fusion can yield measurable improvements in classification performance compared to individual models and prior ensemble strategies, while evaluating their capacity to generalize effectively across heterogeneous chest disease datasets.

The main contributions of this study are listed below:

- Overcoming data leakage: A strict patient-wise split is enforced to prevent images from the same patient appearing in both training and test sets, addressing a known limitation of the Kermany et al. dataset [9,10].
- Developing feature-fusion ensemble models: Complementary deep features from adapted versions of ResNet-50, VGG-19, and MobileNet-V2 are fused after Chi-square feature selection to build more discriminative representations.
- Enhancing model interpretability: Gradient-weighted Class Activation Mapping (Grad-CAM) is applied to localize image regions driving the predictions, improving clinician trust and understanding.
- Robust quantitative evaluation of explainability: Sanity checks based on systematic parameter randomization are conducted to verify that the explanations faithfully reflect the model’s decision-making process.
- Robust evaluation protocol: Single and ensemble models are evaluated using 5-fold cross-validation (CV), independent test (IT) sets, and external lung disease datasets with comprehensive metrics (accuracy, precision, recall, F1-score, specificity, ROC-AUC, PR-AUC, Brier score).

The remaining parts of this article are structured as follows: Section 2 provides a comprehensive overview of related works. Section 3 outlines the methodology. Section 4 presents, compares, and analyzes the classification results. Section 5 compares the proposed approach with successful methods from the literature that used the same dataset and also reports its performance on additional chest disease datasets. Finally, Section 6 concludes the paper and outlines directions for future research.

## 2. Related Research

Recently, DL techniques, particularly CNN-based methods using CXRs, have gained significant interest for classifying chest diseases like pneumonia to improve the effectiveness and validity of clinical care. In the following section, we present some successful models, primarily based on customized architecture, pre-trained networks and ensemble models.

### 2.1. Custom CNN Models

In the case of customized models, researchers in [11,12,13,14] have designed and trained various CNN architectures from scratch. Rajaraman et al. [11] assessed a tailored CNN model built to differentiate normal from pneumonia cases. The suggested model achieves 94.1% accuracy based on the cropped lung ROI representation. In Reference [12], Saraiva et al. investigated pneumonia detection applying CNN and multi-layer perceptron (MLP). The obtained results showed that the custom CNN attained a high accuracy of 94.4%, while the MLP attained 92.16%. Stephen et al. [13] trained a 12-layer CNN on lung X-rays of varying sizes, reaching 93.73% validation accuracy. Rahib et al. [14] compared CNNs with backpropagation neural networks (BPNNs) and competitive neural networks (CpNNs) across 12 chest pathologies, achieving 92.4% validation accuracy. Hosny et al. [15] proposed a multi-modal deep-learning framework for lung cancer detection by integrating an Inception–ResNet backbone with Hierarchical Feature Extraction (HFE) blocks and Efficient Channel Attention (ECA) mechanisms. Their model achieved state-of-the-art performance, reaching 95.35% accuracy on CXR scans, 99.68% on CT scans, and 99.73% on histopathology images. While custom CNNs show promise, their performance is often constrained by dataset size, high computational cost, and absence of robust interpretability.

### 2.2. Transfer-Learning-Based CNN Architectures

To address data scarcity and reduce training cost, transfer learning (TL) has been widely adopted has been widely used. Rajpurkar et al. [16] proposed CheXNet, a 121-layer DenseNet trained on ChestX-ray14, surpassing radiologists in pneumonia detection. Salehi et al. [17] evaluated ResNet-50, Xception, VGG-19, and DenseNet-121 for pediatric pneumonia detection, with DenseNet-121 achieving 86.8% accuracy. In [18], MobileNet achieved the highest accuracy of 92.98% among five pretrained architectures. Kavya et al. [19] compared ResNet-50 and VGG-16, with ResNet-50 outperforming VGG-16 by 2.05% (91.39% accuracy). These studies demonstrate that TL enhances performance, yet the variable performance of individual pre-trained models underscores their limited capacity to extract sufficiently diverse and discriminative features for reliable diagnosis.

### 2.3. Ensemble Models

In recent works, ensemble methods emerged to show enhanced performance and increased classification accuracy. Mittal et al. [20] proposed a hybrid convolutional–capsule network (E4CC), achieving 96.36% accuracy. In [21], features from CheXNet and VGG-19 were combined and reduced via PCA, with Random Forest achieving 98.93% on 5-fold CV. Mabrouk et al. [22] concatenated feature vectors from DenseNet-169, MobileNet-V2, and Vision Transformer (ViT), achieving 93.91% accuracy. Qiuyu An et al. [23] incorporated attention mechanisms into EfficientNet-B0 and DenseNet-121, reaching 95.19% accuracy. Chouhan et al. [24] applied majority voting across five CNN architectures, attaining 96.39% accuracy. Soft-voting ensembles of MobileNet, MobileNet-V2, and DenseNet-169 achieved 96.31% accuracy [25]. Ayan et al. [26] combined the predictions of three top-performing pre-trained networks (ResNet-50, MobileNet, and Xception) selected from a pool of seven candidate models. Their method achieved 95.83% accuracy for binary classification. Additionally, it obtained an accuracy of 90.71% for multi-class classification when distinguishing between normal, bacterial, and viral chest X-rays.

Although ensemble models improve outcomes over single CNNs, most approaches operate at the decision level (voting or averaging) and ignore internal feature representations. This limits achievable accuracy in pneumonia analysis. Some studies explored feature-level fusion [21,22,23,27], but high-dimensional fused features can introduce noise, artifacts, duplicates or unrelated features that can negatively impact network performance.

### 2.4. Summary of Gaps

From the above, three key limitations emerge:

- Single CNNs often capture a narrow set of features, limiting generalization.
- Traditional ensemble approaches ignore feature-level information and may propagate redundancy.
- High-dimensional feature fusion lacks selective mechanisms to retain only the most informative features.

These gaps motivate our Chi-square Selected Feature Fusion Ensemble (CSFFE), which selectively extracts discriminative features from three pretrained CNNs (VGG-19, ResNet-50, MobileNet-V2) before fusion. This approach enhances accuracy, reduces feature redundancy, and provides a compact representation suitable for pediatric pneumonia detection. The distinguishing contributions that differentiate our study are summarized in Table 1.

## 3. Materials and Methods

### 3.1. Data Description

**Figure 1:**
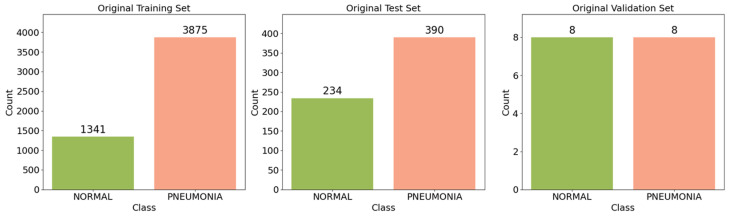
Original distribution of the Kermany dataset.

This study used a collection of 5856 anterior–posterior radiographs of children aged one to five, obtained from the Guangzhou Women and Children’s Medical Center during routine pediatric care. The dataset has been made freely available on Kaggle by Kermany et al. [9,10], where the competition results from various participating teams can be consulted as a reference for performance benchmarks [30]. The dataset contains 1583 normal scans and 4273 pneumonia scans. The pneumonia class includes both viral and bacterial cases, grouped into a single category. Initial data analysis of the CXR was conducted by excluding low-resolution or unreadable radiographs. The integrity of the remaining radiographs was then evaluated as corresponding to infected patient or a healthy subject by two medical specialists. To guarantee the reliability of the annotation procedure, a third expert was also asked to verify the assessment set. The dataset was systematically divided into training, validation, and testing subsets as shown in Figure 1.

### 3.2. Data Splitting

We first examined the original data distribution and identified overlapping patient IDs between the training and test splits. To eliminate potential data leakage, all chest X-ray images were grouped by patient ID, ensuring that images from the same patient were assigned to only one partition. An independent test set corresponding to 10% of the total data was created at the patient level, ensuring that only unseen patients were included.

A fixed random seed (42) was applied during all splitting procedures to guarantee reproducibility. Table 2 reveals data distribution of patients and the corresponding number of images for each class in the training and testing sets.

To further assess robustness and generalization, 5-fold CV was applied within the training portion, with folds defined using the same fixed seed. The training set, corresponding to 90% of the dataset, was divided into five approximately equal parts. Each part served once as a validation fold, while the remaining four were used for training. This process ensured that all samples contributed to both training and validation across iterations, reduced performance variability, and provided a reliable estimate of the model’s effectiveness.

The test set may include multiple images per patient; however, all images belong to patients entirely excluded from training. This design reflects real clinical practice, where multiple views or follow-up images are often available per patient.

### 3.3. Data Preprocessing

**Figure 2:**
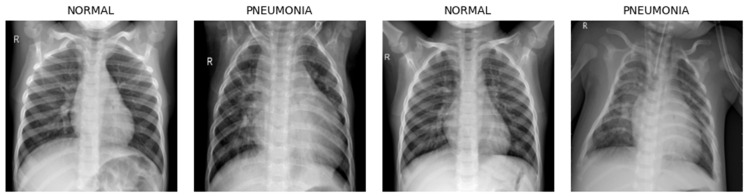
Examples of pediatric CXRs samples with and without pneumonia conditions with a resolution of 150 × 150.

The original pediatric radiographs in the Kermany dataset are provided in varying dimensions. Given this heterogeneity, these images are resized to 150 × 150. This reduction is employed to accelerate the learning process and also decrease memory usage. The Comparison of different input image resolutions in terms of balancing diagnostic feature retention and computational demand are provided in Section 5.1. Figure 2 shows some random examples of radiographic images. After resizing, the pixel values of the images were normalized from their original 0–255 range to 0–1 to standardize the data on a similar scale without losing important information. Since pretrained networks such as VGG-19, ResNet-50, and MobileNet-V2 are designed for color image classification, the images were transformed into RGB channels to be compatible with these models and to ensure better feature extraction.

### 3.4. Data Augmentation

In this study, data augmentation (DA) was applied after splitting and preprocessing using ImageDataGenerator from the Keras library exclusively during the training process. This step is crucial to reduce the risk of overfitting in each epoch and to make the model robust to spatial and orientation variations. For the test set, the DA method was not applied to ensure that the system is evaluated only on original data.

The process involved a series of geometric transformations, starting with rotation, followed by shear and zoom, and concluding with a horizontal flip. Details of the specific transformations, their parameters, and clinical relevance are summarized in Table 3.

### 3.5. Deep Feature Extraction Using Pretrained Networks

The effectiveness of CNNs relies on the available training data. However, this is not usually the case in the medical field, where obtaining sizable, labeled datasets for training is impractical due to privacy concerns and the high costs of expert annotations. Transfer learning and fine-tuning (FT) can be employed to address this issue [31].

According to [32], the pre-trained models ResNet-50, VGG-19, and MobileNet-V2 have achieved over 92% accuracy in chest disease detection. Based on these reports, these models were selected as pretrained backbones for deep feature extraction within a transfer learning setting. In addition, this choice is closely related to the characteristics of the Kermany dataset [9,10], which is relatively small and exhibits a certain degree of class imbalance. In such conditions, excessively deep or highly complex architectures tend to overfit and often require aggressive fine-tuning and strong regularization to generalize properly. In contrast, ResNet-50, VGG-19, and MobileNet-V2 have consistently demonstrated stable and reliable performance on limited-scale medical imaging datasets. Therefore, these architectures were intentionally chosen to provide robust and well-established feature extractors, allowing the evaluation to focus primarily on the effectiveness of the proposed feature-level fusion strategy rather than on architectural complexity.

#### 3.5.1. Fine-Tuning Strategy and Ablation Analysis

To adapt the pretrained backbones to the pneumonia classification task while preventing overfitting, a structured TL strategy was adopted rather than arbitrarily freezing or unfreezing layers. Initially, the original classification heads of ResNet-50, VGG-19, and MobileNet-V2 were replaced with task-specific fully connected layers, and all convolutional layers were frozen to enable the newly added classifier to learn pneumonia-related patterns. Subsequently, progressive FT was performed by gradually unfreezing deeper portions of each backbone. Multiple configurations were systematically evaluated, including fully frozen networks, partial unfreezing of the last 5, 15, and 45 layers, and full end-to-end training. The final configuration for each backbone was selected based on a favorable trade-off between meaningful improvements in 5-fold CV performance, stable generalization, and training efficiency. As shown in Table 4, the results highlight the influence of progressive layer unfreezing during the fine-tuning process. Starting from fully frozen backbones (0 trainable layers), the models exhibit limited adaptation, as the extracted features mainly reflect generic ImageNet representations. Gradually unfreezing additional layers (5 and 25 layers) allows the networks to better adapt to pneumonia-specific characteristics, leading to improvements in validation accuracy and reduced loss.

**Figure 3:**
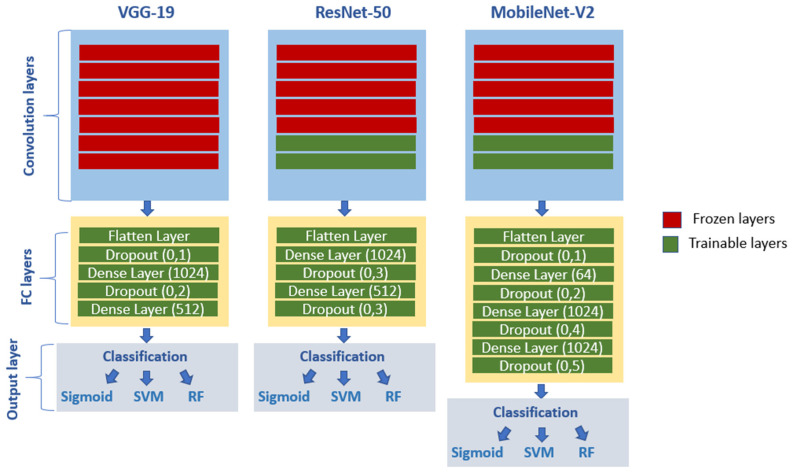
Configuration details for transfer learning and fine-tuning strategies for individual models.

However, when all layers are unfrozen, performance begins to decline and the loss increases, likely due to overfitting and the increased difficulty of optimizing a large number of parameters on a relatively small and imbalanced dataset. Furthermore, the results reveal a clear relationship between the number of trainable parameters and training time: deeper fine-tuning substantially increases computational cost. Although retraining 25 layers yields the highest accuracy, the improvement remains marginal compared to the additional runtime. Therefore, retraining only the last five layers for ResNet-50 and MobileNet-V2, and keeping VGG-19 fully frozen, provides a more favorable trade-off between accuracy, generalization, and computational efficiency. The FT configurations are shown in Figure 3.

#### 3.5.2. Hyperparameter Tuning

Grid search is among the most commonly employed techniques for hyperparameter tuning. Despite its high computational demand, this exhaustive strategy systematically evaluates all possible parameter combinations to determine the best-performing configuration without manual intervention. Considering the relatively small size of our dataset, this method was used to identify the optimal hyperparameter settings for both single and ensemble models. It was preferred despite its computational cost, as it provides complete coverage of the hyperparameter space, ensures reproducibility, and increases the likelihood of finding the global optimum compared to probabilistic or population-based alternatives.

Each parameter range was defined based on existing literature and empirical results from prior studies. All experiments were performed using a fixed random seed (42) to guarantee reproducibility, and the best-performing configuration was retrained from scratch during each fold of the 5-fold CV. Table 5 summarizes the optimal hyperparameters and their tested intervals.

### 3.6. Discriminative Localization with GRAD-CAM for Visual Explanation

While CNNs have attained strong results in various tasks, particularly in image classification, there are still many essential questions referring to the reason and manner of such outstanding achievements. The “black box” nature of CNNs makes them difficult to apply, especially in scenarios where interpretability is crucial, such as in medical diagnosis applications. Grad-CAM addresses this challenge by visually highlighting the most important regions of an image on which a model relies for its predictions. It achieves this by tracing the gradients of the predicted class back to the final convolutional layer, hence generating a heatmap that shows informative regions used by the model for classification.

In this study, the Grad-CAM algorithm is integrated with the proposed models to locate the decision area on a heatmap. Pixels with stronger gradients are represented by warmer colors, such as red and yellow, while areas with weaker gradients are shown in cooler tones, like green and blue.

#### Sanity Check

To verify the reliability of the Grad-CAM visual explanations, a sanity check was conducted following the approach introduced by Adebayo et al. [33]. A dependable XAI technique should respond to the model’s learned parameters, which capture the knowledge obtained during training. We applied cascading model randomization to VGG-19, ResNet-50, and MobileNet-V2. For each model, the weights of selected convolutional layers were gradually randomized from the uppermost layers toward the lower ones. Grad-CAM heatmaps were generated for the original trained model and after each randomization stage.

The Structural Similarity Index Measure (SSIM) [34] was calculated between the baseline heatmap and each randomized heatmap to evaluate the influence of parameter disruption. SSIM is constructed to replicate human visual perception of likeness between images by considering structural composition, brightness, and contrast variations. In contrast to traditional error-based measures such as mean squared error, SSIM functions as a perception-oriented evaluation that aligns more closely with the cognitive interpretation of the human eye. A clear reduction in SSIM across randomization levels confirmed that Grad-CAM visualizations depend on the learned representations of each network rather than on architectural or preprocessing factors. This validation indicates that the produced heatmaps deliver reliable and model-dependent insights into the visual attributes driving pneumonia detection. The findings are provided in Section 4.3.2.

### 3.7. Feature Selection with Chi-Square Test

The procedure of feature selection (FS) is an essential phase to enhance classification success in DL models. The main aim of this method is to identify and use the most informative features while discarding redundant ones from the initial set of extracted features. This study employed the filtering method to achieve feature scoring based on the non-parametric Chi-square test. The flattened layer output is given to the Chi-2 algorithm [35] to identify the top k informative patterns and minimize bias during training. As a result, it is anticipated to achieve superior outcomes by combining highly informative features.

The Chi-2 algorithm is used to assess the independence of each feature with the output variable and produces a score reflecting the strength of their association. A higher Chi-2 score suggests that the feature is highly correlated with the predicted category.

In a two-class classification task, the Chi-2 method arranges the input variables by importance as follows: considering a total of (t) instances and a pair of class outputs, positive and negative. Table 6 can be designed to calculate the ${ꭓ}^{2}$ test score. $p_{1}$ and $n_{1}$ represent, respectively, the numbers of positive and negative instances when the feature ($x_{i}$) is active, while $p_{2}$ and $n_{2}$ represent, respectively, the counts of samples labeled as positive and negative when the feature ($x_{i}$) is inactive. *p* indicates the total count of positive instances, and $\left(t-p\right)$ corresponds to the total count of negative instances. On the other hand, m and $\left(t-m\right)$ refer to the total count of instances when the feature ($x_{i}$) is active and inactive, respectively.

Based on the total count of data points and the distribution of positive and negative outcomes, the ${ꭓ}^{2}$ test measures the divergence between the expected values ($E_{i}$) and the observed values ($O_{i}$), using Equation (1) from which the specific form in Equation (2) is derived. The expected value $E_{p1}$ is computed using the formula in Equation (3); similarly, the other expected values $E_{n1}$, $E_{p2}$, $E_{n2}$ are computed.

The calculated ${ꭓ}^{2}$ score is then compared to the Chi-2 distribution table with the degrees of freedom (df = 1) to determine statistical significance. If the computed ${ꭓ}^{2}$ exceeds the threshold, then the *p*-value is smaller than 0.05, we reject the null hypothesis; in other words, the feature correlated with the output and should be retained. Conversely, a low value suggests the feature is not significantly contributing to the classification task and can be excluded.

(1) $${ꭓ}^{2}=\sum \frac{{\left(O_{i}-E_{i}\right)}^{2}}{E_{i}}$$

(2) $${ꭓ}^{2}=\frac{{\left(O_{p1}-E_{p1}\right)}^{2}}{E_{p1}}+\frac{{\left(O_{n1}-E_{n1}\right)}^{2}}{E_{n1}}+\frac{{\left(O_{p2}-E_{p2}\right)}^{2}}{E_{p2}}+\frac{{\left(O_{n2}-E_{n2}\right)}^{2}}{E_{n2}}$$

(3) $$E_{p1}=\frac{m\times p}{t}$$

### 3.8. Feature Fusion

Feature Fusion (FF) refers to merging diverse features extracted from different models or modalities to create the best single feature set. The primary objective of fusion data in various medical applications is to combine the discriminative complementary features into a single feature set used as input for the subsequent phase in the workflow. This aims to boost performance and reduce training time.

The success of the FF approach relies on the selection process of the more appropriate features to accurately detect the disease and significantly enhance the classification accuracy. Standard methods for FF include concatenation, addition, and multiplication.

The present study used horizontal feature concatenation due to its straightforward implementation and its ability to preserve the individual contribution of each model. Once the most pertinent features for classification were identified from each CNN model using SelectKBest with the Chi-2 test, they were normalized using the Z-score to ensure consistent feature scaling. The normalized feature sets were then fused using four different combinations. Firstly, all SelectKBest features from the three models (VGG-19, ResNet-50, and MobileNet-V2) were concatenated. Then, the selected Chi-2 features from two models (VGG-19 and ResNet-50) were combined. After that, the selected feature vectors from the fine-tuned versions of (ResNet-50 and MobileNet-V2) were concatenated. Lastly, the most informative features from (VGG-19 and MobileNet-V2) were fused to form the fourth combination. The combined configurations were then used as input for various classifiers in the detection stage to identify the most effective hybrid configuration.

### 3.9. Pneumonia Detection

Various classification algorithms were employed in this research to classify the pneumonia disease using concatenated Chi-2 features.

#### 3.9.1. Sigmoid Classifier

In this work, callbacks were added to the single and ensemble models training with FC layers to prevent overfitting. For the ReduceLROnPlateau callback, the LR was decreased by 0.2 when validation loss did not improve over three consecutive epochs. In addition, the EarlyStopping callback was added to the model training with 5 patience.

The grid search yielded the following hyperparameter values for training the hybrid ensemble models with FC layers: a batch size of 32, 25 epochs, an LR of 1 × 10^−4^, a dense layer with 512 neurons that used ReLU activation, and a single dropout of 20%. The final layer used a sigmoid function to perform the two-category classification. The models were compiled with the Adam optimizer to minimize BCE loss, with accuracy as the primary assessment metric.

#### 3.9.2. SVM Classifier

To train the combined features from two and three CNNs using the SVM classifier, the hyperparameters needed to be determined first. To this end, different values of the kernel, gamma and the penalty parameter C were considered. The values that produced the highest validation accuracy were the linear kernel and C = 1. Additionally, the random state was fixed at 42 to ensure the reproducibility of the results.

#### 3.9.3. RF Classifier

To train the combined features from two and three CNNs using the RF classifier, the hyperparameters needed to be tuned first. To this end, different values of n_estimators, min_samples_split and max_depth were considered. The values that produced the highest validation accuracy were an ensemble of 500 DT, min_samples_split of 2 and a max depth of 10. Additionally, the random state was fixed at 42 to ensure the reproducibility of the results.

### 3.10. The Proposed CSFFE Approach

**Figure 4:**
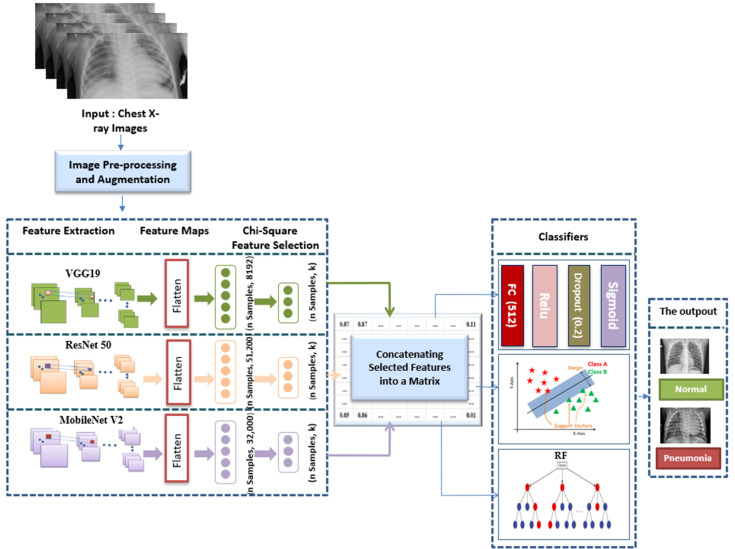
The visual procedure of the suggested methodology for childhood pneumonia detection.

The goal of the recommended CSFFE framework is to enhance pneumonia detection using the most suitable and strongly associated feature sets extracted by three widely recognized CNN models. Figure 4 presents the visual representation of the suggested pneumonia recognition approach and highlights the design employed in this approach.

The suggested approach begins by preprocessing the provided chest radiographs prior to providing them to the CNN models for learning. The process then moves forward in two separate phases. During the initial classification phase, an attempt was undertaken to examine three CNN configurations and their hyperparameters, seeking to determine the single model that provided optimal results for pneumonia identification. These individual models serve as tools for extracting features through TL and a shallow tuning strategy to classify lung scans as pneumonia or normal using different classification algorithms. In the next classification step, deep features extracted from the flatten layer of each backbone network are directly subjected to a Chi-2 test, to select the most relevant ones. The Chi-2 was applied to test several values of k features from those initially extracted by each model, and the best value was selected based on 5-fold CV accuracy. Following that, the chosen features corresponding to the optimal k were horizontally combined together in various combinations. The resulting CSFFE matrices were then paired with FC, SVM, and RF classifiers to automatically classify pediatric scans into “Pneumonia” and “Normal”.

#### Novelty and Methodological Justification

Detecting illnesses such as pneumonia from limited and biased thoracic chest X-ray datasets remains challenging, and demands robust methodological solutions that enhance feature discrimination while controlling model complexity. The proposed work introduces a novel feature-level fusion framework termed CSFFE, which distinguishes itself via the exploitation of complementary deep representations learned by ResNet-50, VGG-19 and MobileNet-V2. Unlike conventional ensemble approaches that operate exclusively at the decision level by averaging probabilities or applying majority voting, CSFFE performs fusion at the feature level. This design explicitly exploits the integration of heterogeneous deep representations prior to classification and allows the subsequent learning stage to exploit complementary information across models within a single feature space.

The CNN architectures used in this study rely on common core components, including convolutional layers, pooling operations, and regularization mechanisms. Each model introduces distinct structural design choices that influence feature extraction behavior, computational complexity, and training stability:

- VGG-19 [36] represents a deep plain convolutional architecture with uniform 3 × 3 convolutional filters and a large number of parameters, providing stable and well-structured feature maps.
- ResNet-50 [37] introduces residual skip connections that facilitate gradient propagation in deeper networks and enable the learning of hierarchical representations with increased depth.
- MobileNet-V2 [38] adopts depthwise separable convolutions and linear bottleneck blocks, resulting in a lightweight architecture with reduced computational cost and compact feature representations.

This structural diversity introduces heterogeneity in network depth, connectivity patterns, and parameterization, which is particularly suitable for feature-level fusion within the proposed CSFFE framework. Table 7 summarizes the main architectural and computational characteristics of the backbone networks used in this study.

The core innovation lies in the integration of a Chi-square-based statistical feature selection stage prior to feature fusion, which retains only the most discriminative components from each model. This step effectively reduces redundancy and dimensionality, mitigates overfitting, and enhances the discriminative power of the fused feature vector, making the framework well suited for small and highly imbalanced pediatric datasets. Experimental results demonstrate that CSFFE consistently outperforms individual CNN models and conventional ensemble methods while maintaining low computational cost and good generalization across different chest diseases under highly imbalanced class settings. Thus, the contribution of this work is not in using well-established architectures separately but in a statistically guided ensemble-driven design, which remains underexplored in pediatric chest X-ray analysis and provides a reliable solution for automated diagnosis.

### 3.11. Experimental Setup and Evaluation Metrics

This work was conducted online using the Kaggle platform with a P100 GPU to speed up the execution of our code. For building and training the proposed models, the software environment included Python 3.10.13, Keras 3.3.3, and TensorFlow 2.15.0.

The evaluation of single and feature fusion-based ensemble models utilizing DL and ML algorithms with CXR images encompasses the assessment of two principal dimensions: discriminative ability and calibration reliability.

Discriminative ability evaluates how effectively the model separates positive and negative cases. It was assessed using complementary threshold metrics, including accuracy (Acc), precision (Pre), recall (Rec), specificity (Spe), and F1-score (F1), as well as ranking metrics, such as Area Under the Receiver Operating Characteristic Curve (ROC-AUC) and Precision–Recall AUC (PR-AUC).

Threshold metrics evaluate classification errors by comparing predicted labels with actual outcomes, measuring performance at a fixed decision boundary. On the other hand, ranking metrics quantify the model’s ability to consistently assign higher scores to positive instances than to negative ones.

The values of the aforementioned threshold parameters were calculated using the confusion matrix. This method is the principal way to visualize the number of true positives (TP), true negatives (TN), false positives (FP), and false negatives (FN). These metrics are formally defined as:

(4) $$Acc\;=\;\frac{\left(TP+TN\right)}{\left(TP+TN+FP+FN\right)}$$

(5) $$Pre=\frac{TP}{\left(TP+FP\right)}$$

(6) $$Rec\;\left(Sensitivity\right)=\frac{TP}{\left(TP+FN\right)}$$

(7) $$Spe=\frac{TN}{\left(TN+FP\right)}$$

(8) $$F1=2\times \frac{Pre\times Rec}{\left(Pre+Rec\right)}$$

The prediction outcomes were visualized using ROC curves, which illustrate the trade-off between true positive and true negative rates across all decision thresholds. For better classification results, the ROC curve should approach or reach the top-left corner. The AUC score, derived from the ROC curve, ranges from 0 to 1 and reflects the model’s ability to distinguish between the two classes. An AUC value of 1 indicates perfect separation, while a value of 0 means the model completely confuses healthy and pneumonia cases.

While ROC curves provide an overall measure of classification performance, they can be misleading on imbalanced datasets by favoring the majority class. In such cases, precision–recall (PR) curves are more appropriate, plotting precision against recall and focusing on positive class detection. The PR-AUC summarizes the model’s ability to identify positives while minimizing false positives, which is critical for detecting rare classes.

Calibration reliability measures the agreement between predicted probabilities and actual outcomes. It was evaluated using the Brier score (BS) and calibration curves. The BS is a reliable scoring method that calculates the mean squared difference between the predicted probability for each observation ($p_{i}$) and its actual binary outcome ($o_{i}$) over all N samples. It offers an overall assessment of both predictive accuracy and calibration. Smaller Brier scores indicate models whose predicted probabilities closely align with the true outcomes, reflecting better calibration. Calibration plots were additionally used to visualize this agreement across the range of predicted probabilities.

(9) $$BS=\frac{1}{N}\overset{N}{\underset{i=1}{\sum }}{\left(p_{i}-o_{i}\right)}^{2}$$

These measures together offer a thorough evaluation of the model’s performance, extending beyond simple accuracy. They account for the balance between sensitivity and specificity, the trade-offs between precision and recall, and the reliability of predicted probabilities through calibration.

During training, performance metrics were calculated for each fold, and the final CV results were obtained by averaging the outcomes from all five validation sets. After completing the training process, the same models were also tested on the IT set to assess their practical effectiveness.

## 4. Results

This section will analyze and compare the performance metrics used in this work in order to detect pneumonia disease in two distinct datasets (CV and IT sets) using individual and feature fusion-based ensemble models.

### 4.1. Performance of Single DL Models

The goal of this initial classification experiment is to assess the effectiveness of deep patterns derived from the modified pre-trained models. The experiment results on the CV and IT sets are displayed in Table 8 and Table 9. According to the mean CV, and IT results on Kermany dataset, all three models demonstrated good performance, exceeding 93% accuracy across all classifiers for binary classification, which proves that the extracted deep features are highly discriminative and generalize well.

In addition, our classification experiment underscores the considerable influence of classifier selection on the model’s efficacy. As can be seen, among the evaluated classifiers on both CV and IT datasets, SVM outperforms the others in most cases. It consistently achieves the highest accuracy, recall, Specificity, and lowest Brier score, indicating both strong predictive accuracy and better probability calibration. The FC classifier also delivers solid results, with accuracy, precision and AUC values close to those of SVM, particularly when combined with VGG-19 and MobileNet-V2. Its good balance between sensitivity and specificity shows that it generalizes well to unseen data. Conversely, the RF classifier shows the lowest performance among the three. It provides good recall but consistently lower specificity and higher Brier scores, meaning it tends to produce more false positives and less calibrated outputs.

According to the obtained findings, VGG-19 model consistently outperformed the other models during 5-fold CV. When combined with the SVM classifier, it reached the highest average accuracy of 96.33% (95% CI: 95.65–97.01), confirming stable performance across folds. Its mean precision of 97.20% (95% CI: 96.38–98.02) indicates that almost all predicted pneumonia cases were correctly identified. The F1-score, which balances precision and recall, reached 97.52% (95% CI: 97.06–97.98) reflecting a strong equilibrium between detection and diagnostic reliability. The model also obtained the lowest Brier score of 2.85% (95% CI: 2.56–3.26).

In addition, when coupled with FC classifier, VGG-19 achieved an even higher recall of 98.67% [95% CI: 97.52–99.82], the best among all models, highlighting its superior sensitivity in detecting pneumonia cases. It also maintained top AUC (99.20% (95% CI: 98.94–99.46)) and PR-AUC (99.70% (95% CI: 99.58–99.81)) values, indicating excellent discrimination between positive and negative classes. These results demonstrate a strong balance between sensitivity and precision, confirming that VGG-19 features are highly discriminative and effective for pneumonia classification.

On the independent test set, MobileNet-V2-based models achieved the best overall performance across all evaluation metrics, confirming their strong generalization and effective feature extraction ability. When combined with the SVM classifier, MobileNet-V2 reached the highest accuracy (95.74%), precision (95.90%), and specificity (91.76%), along with a competitive F1-score (96.84%). It also achieved the lowest Brier score (2.93%), reflecting well-calibrated probability estimates, and demonstrated an excellent AUC (99.42%) and PR-AUC (99.71%), highlighting its strong discriminative power. These results show that MobileNet-V2 provides a balanced trade-off between sensitivity and precision, confirming its reliability and robustness for pneumonia detection on unseen data.

Modified ResNet-50 demonstrated strong performance on both datasets, with only minor variations. Mean Accuracy ranged from 95.15% (RF) to 96.16% (SVM) on the CV dataset and from 94.63% (RF) to 95.56% (SVM) on the IT dataset. It also maintained balanced performance, with stable F1, AUC, and specificity values, reflecting a good compromise between the detection of positive and negative samples.

Overall, these findings confirm that SVM and FC classifiers outperform RF when applied to deep feature representations extracted from VGG19, ResNet50, and MobileNetV2. The results also highlight that VGG-19 and MobileNetV2 represent the most effective single models.

### 4.2. Grad-CAM Visual Insights of Models Predictions

**Figure 5:**
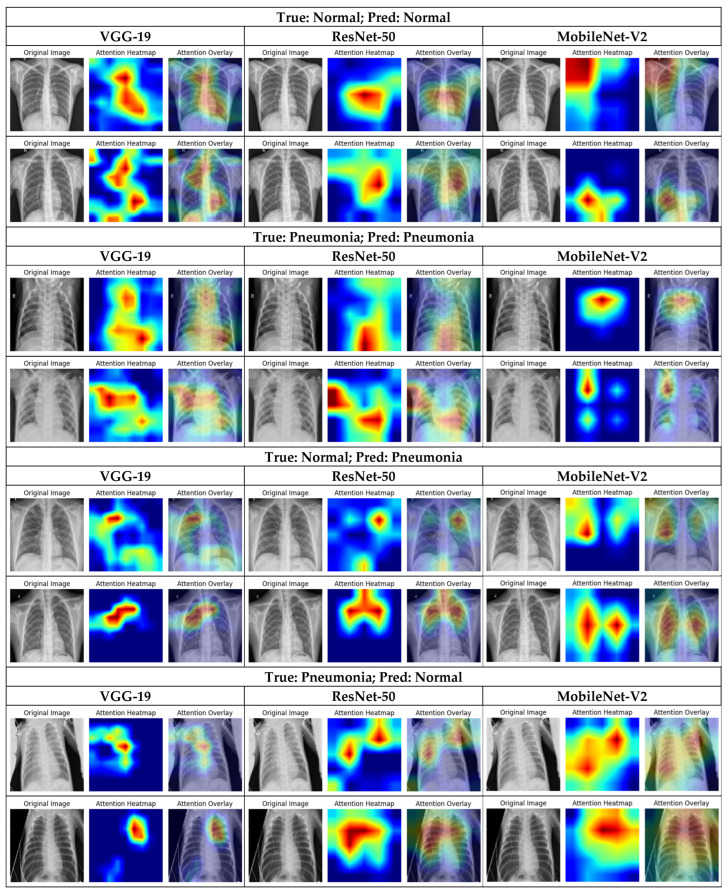
Grad-CAM visualizations of correctly and incorrectly classified samples by VGG-19, ResNet-50, and MobileNet-V2, highlighting the salient regions influencing model predictions.

Figure 5 presents the results of the Grad-CAM analysis obtained from healthy and diseased lung X-rays samples. Clearly, the models effectively targeted the lung area to detect pneumonia, and interestingly, they highlight different salient regions from the same input radiographs. This demonstrates the complementary nature of the features learned by each architecture and supports their combination in ensemble models to enhance classification performance.

The Grad-CAM heatmap was generated by performing a forward pass through the model to extract feature maps, followed by a backward pass to compute the gradients of the target class with respect to these maps. These gradients were globally averaged to obtain channel-wise weights, which were then combined with the feature maps to produce the raw attention map. After normalization and resizing, the heatmap was superimposed on the original X-ray image to visualize the regions of focus.

Figure 5 shows the Grad-CAM analysis for the baseline models on chest X-rays. For each model, two correctly predicted normal cases and two correctly predicted pneumonia cases are displayed. The original image is shown alongside its corresponding heatmap and the overlaid image, where the highlighted regions indicate the areas that are most influential for the model’s prediction. Similarly, two misclassified normal cases (predicted as pneumonia) and two misclassified pneumonia cases (predicted as normal) are shown for each model. The heatmaps for these incorrectly predicted samples reveal the regions that led the model to make errors, providing insight into the specific features or areas that the model misinterpreted. Visualizing Grad-CAM in these cases helps understand why the model failed, highlighting potential confounding patterns or regions that contributed to the misclassification, and offering guidance for improving feature extraction or model training. These visualizations provide insight into both successful predictions and the causes of misclassification, emphasizing the complementary features captured by different baseline models.

Based on these observations, we suggest that combining deep features extracted from the considered models allows the integration of diverse feature representations learned by each CNN. In addition, applying the Chi-2 algorithm to select the most relevant feature subsets from each model can significantly improve sensitivity and specificity for detecting pathological patterns while reducing the risk of missing critical abnormalities.

#### 4.2.1. Correctness—Sanity Check of Grad-CAM Visualizations

We applied the cascading model randomization sanity check introduced by [33] to evaluate whether Grad-CAM explanations truly depend on the learned parameters of each CNN. This test verifies that the visual explanations are sensitive to model weights updated during training. If the generated heatmaps remain unchanged after weight randomization, it indicates that Grad-CAM is not responding to learned representations and therefore is unreliable. Passing the sanity check is therefore essential to confirm that the explanations depend on the model’s training rather than its structure alone. However, this procedure only verifies their validity, not their internal consistency or interpretive coherence. Since the sanity check verifies model reliability and requires intensive computation, we applied it only to the same correctly classified chest X-rays used for Grad-CAM visualization, including two normal and two pneumonia cases per model.

**Figure 6:**
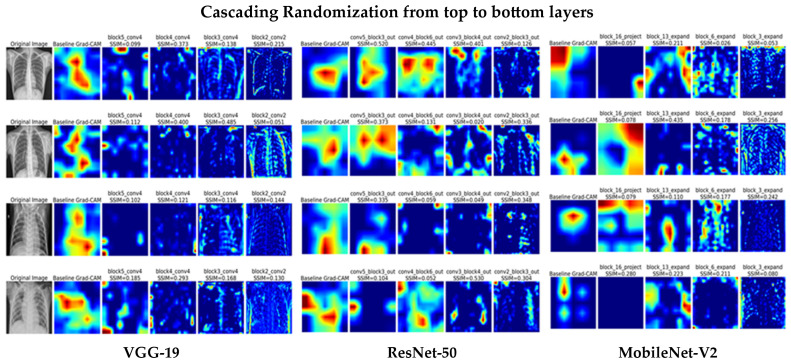
Grad-CAM sanity check: attention maps from VGG-19, ResNet-50, and MobileNet-V2 with cascading model randomization on normal and pneumonia chest X-rays.

As described previously in Section Sanity Check, this procedure involved sequential randomization of convolutional layer weights from the top to the bottom layers, starting with the highest-level feature maps. Figure 6 presents the new Grad-CAM heatmaps generated after each randomization and compared with the baseline using SSIM. The resulting explanations showed clear visual differences from those of the original model. The study [33] reported that Guided Backpropagation failed this test, as its SSIM values remained close to 1.0 even after repeated randomization of higher-level layers, indicating limited sensitivity to model parameters.

For VGG-19, the SSIM values decreased notably across all randomized layers, ranging from 0.09 to 0.48 across the four test images. The largest drops occurred after randomizing block5 and block3, showing that Grad-CAM is highly sensitive to parameter changes across both top and deeper convolutional layers. In ResNet-50, SSIM scores fell from around 0.5 in the top layers to below 0.15 in the lower layers, with small fluctuations at intermediate stages, confirming that Grad-CAM responds to changes in model parameters. For MobileNet-V2, SSIM values varied between 0.05 and 0.43 and mostly stayed below 0.28 across all randomization stages, indicating strong sensitivity of the attention maps to weight perturbations.

Taken together, the consistent drop in SSIM across all models and images shows that Grad-CAM reliably reflects the learned representations and successfully passes the sanity check.

#### 4.2.2. Clinical Validation of Grad-CAM Heatmaps

To examine the behavior of the CNNs more closely, we evaluated whether the regions highlighted by our Grad-CAM maps correspond to reported radiological findings. For this purpose, we processed new CXR samples annotated by experts through the proposed models. These images were obtained from [39], which describes the radiological manifestations of tuberculosis. They provide information on the location of specific radiological signs and, where available, their underlying causes. The selected images include arrows indicating key radiological features such as airway involvement, consolidations, and cavitary lesions, which could influence the resulting Grad-CAM heatmaps.

**Figure 7:**
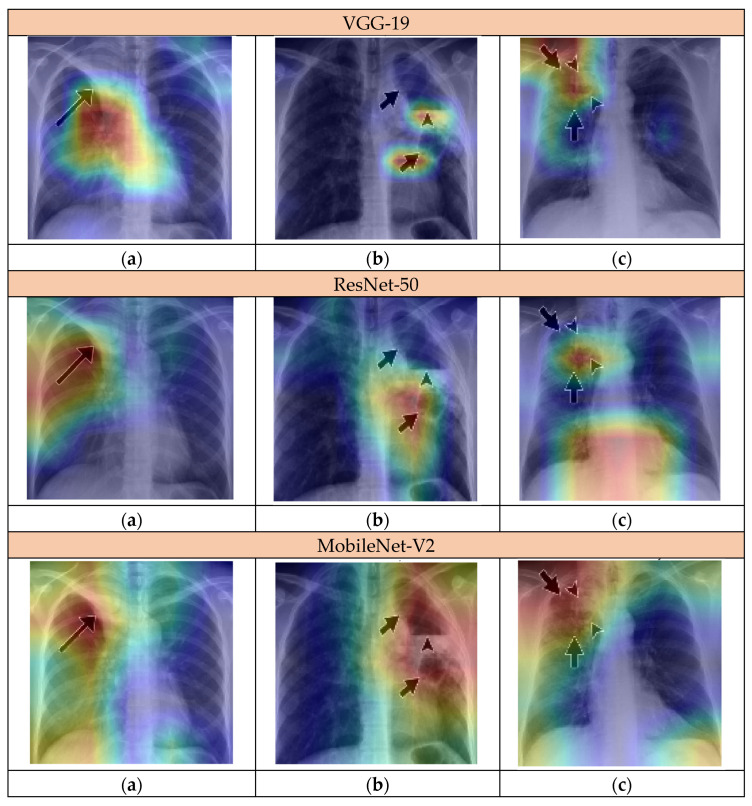
Grad-CAM visualizations on tuberculosis radiographs with expert-annotated radiological findings. (**a**) A 41-year-old woman with airway involvement due to primary tuberculosis. The arrow indicates a right upper lobe collapse caused by proximal airway obstruction; (**b**) A patient with two cavitary lesions in the left lung (large arrows) and an air–fluid level inside the larger lower cavity (small arrow); (**c**) A 50-year-old patient with post-primary tuberculosis, showing characteristic radiological findings.

The first chest radiograph (Figure 7a) shows a 41-year-old woman with airway involvement due to primary tuberculosis. The arrow indicates a right upper lobe collapse caused by proximal airway obstruction. Grad-CAM results show that all models identify this region. VGG-19 highlights both the bronchial area and adjacent parenchymal consolidation. ResNet-50 captures the bronchial thickening with broader activation extending to the mediastinum. MobileNet-V2 provides the most focused attention on the central bronchial region, accurately localizing the airway involvement.

The three models complement each other with distinct yet complementary feature sensitivity, collectively identifying the tuberculosis-related airway involvement and the radiological signs described in [39].

In the second chest radiograph (Figure 7b), the large arrows indicate two cavitary lesions in the left lung, and the small arrow marks an air–fluid level inside the larger lower cavity. VGG-19 and ResNet-50 show distinct focus patterns on this radiograph of a 32-year-old man with post primary tuberculosis. VGG-19 clearly detects the lower cavity and the air–fluid level, showing strong attention to the most active lesion. It fails, however, to capture the upper cavity, missing part of the disease extent. ResNet-50 also targets the lower cavity as the main abnormality but gives limited attention to the air–fluid level and overlooks the upper lesion. Both models identify the primary active area but miss secondary cavitary involvement described in the study. In contrast, MobileNet-V2 successfully identifies the entire pathological region. It captures the upper lesion missed by the other two models and maintains strong activation over the lower cavity and its air–fluid level, demonstrating sensitivity to both primary and secondary disease sites. Together, the three models complement each other. This broader focus compensates for the limitations of VGG-19 and ResNet-50, providing more complete spatial coverage of cavitary involvement and aligning with the radiological description of multiple cavities in the study.

The last chest radiograph (Figure 7c) corresponds to a 50-year-old patient with post primary tuberculosis, showing the typical upper-lobe involvement and cavitary formation of active disease. The large arrows indicate irregular air-space opacities in the right upper lobe representing areas of consolidation, while the arrowheads and small arrow mark a thick-walled cavity within this consolidated zone, consistent with active cavitary lesion. VGG-19 shows intense activation over the right upper lobe, precisely over the cavity and part of the adjacent consolidation, confirming recognition of the main pathological focus. However, its attention is mainly confined to the central and upper part of the lesion, leaving peripheral consolidation underrepresented. ResNet-50 also highlights the upper-lobe cavity but extends activation to the lower lung and mediastinal regions not described in the study, reflecting possible nonspecific activation. In contrast, MobileNet-V2 provides a concentrated response over the entire pathological area, clearly delineating both the cavity and the surrounding consolidation with minimal attention to normal regions.

This initial clinical validation shows that the proposed models can identify the primary pathological regions in tuberculosis chest radiographs, yet some secondary anomalies remain underrepresented. These unnoticed anomalies may result from the presence of arrows in the images, overlapping abnormalities, or limited image quality. The complementary attention patterns of VGG-19, ResNet-50, and MobileNet-V2 enable broader coverage of lesions than any single model alone, partially mitigating these limitations.

To fully capture all relevant features, future work should involve more targeted training supported by qualified clinical experts, such as radiologists and physicians, to guide and validate the models’ attention and enhance detection coverage.

### 4.3. Performance of Feature Fusion-Based Ensemble Models

This section reports the experimental findings of ensemble learning using the most informative feature sets selected from CNNs to evaluate whether combining these optimal features improves classification performance. During this phase, the FS approach was applied separately using the Chi-2 test to the feature representations obtained from each network: 8192 features from VGG-19, 51,200 from ResNet-50, and 32,000 from MobileNet-V2. A set of ten candidate values ranging from 100 to 1000 was evaluated. For each candidate *k*, the top k features based on their ${ꭓ}^{2}$ scores were selected from each CNN, then concatenated horizontally to form the combined feature matrix. The resulting fused subset was used to train and validate the classifier using 5-fold cross-validation. Performance metrics were computed for each fold and averaged to obtain a stable performance estimate. The value of *k* that achieved the highest mean CV performance was identified as the optimal feature subset size. This subset was then used to evaluate the final model on the independent test set.

**Figure 8:**
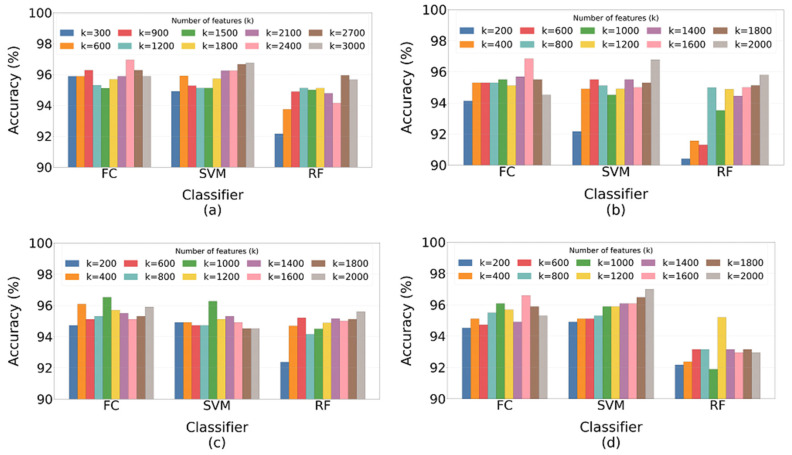
Comparison of different classifiers on feature sets extracted from CNN models: (**a**) VGG-19, ResNet-50, and MobileNet-V2; (**b**) VGG-19 and ResNet-50; (**c**) ResNet-50, and MobileNet-V2; (**d**) VGG-19, and MobileNet-V2.

Figure 8a–d illustrate the evolution of model accuracy as a function of the total number of selected features for different two- and three-model fusion combinations using different classifiers.

Feature-level fusion ensemble models were first assessed using five-fold cross-validation. The results are presented in Table 10, showing the mean values and corresponding 95% confidence intervals for all performance metrics across the folds. The independent test results are summarized in Table 11.

On the cross-validation dataset, the best feature-fusion ensemble achieved a 0.75% improvement in accuracy after applying Chi-squared feature selection and feature concatenation, compared to the best-performing single model from the initial phase (VGG-19 + SVM). Similarly, on the independent test dataset, the best ensemble achieved a 1.85% increase in accuracy over the strongest single model (MobileNet-V2 + SVM), confirming the effectiveness of multi-model feature fusion in improving generalization and predictive reliability.

During five-fold cross-validation, the SVM classification approach reached a mean accuracy of 97.08% (95% CI: 96.71–97.45) using the reduced feature representation obtained by fusing the top 1000 attributes from VGG-19 and MobileNet-V2. The ensemble also achieved a mean precision of 97.83% (95% CI: 97.11–98.55) with minimal variation across folds, confirming its strong capability to correctly identify pneumonia cases. For pneumonia detection, the model attained a mean recall (sensitivity) of 98.20% (95% CI: 97.54–98.86) and a mean specificity of 93.93% (95% CI: 91.83–96.03), indicating balanced and reliable diagnostic performance.

**Figure 9:**
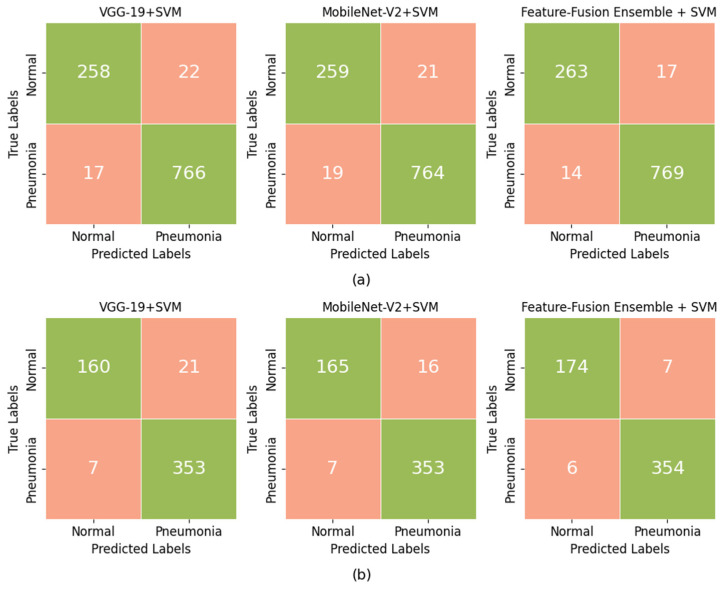
Confusion matrices of SVM classifiers using feature sets from VGG-19, MobileNet-V2, and their fusion: (**a**) Results on CV data; (**b**) Results on the IT set.

As shown in Table 11, the proposed feature-level fusion ensemble of VGG-19 and MobileNet-V2 achieved the highest accuracy of 97.59% on the independent test dataset when combined with the SVM classifier, demonstrating strong generalization capability. This combination also attained a precision of 98.06%, with only seven false positives (see Figure 9), confirming that nearly all positive predictions were correct. This level of precision is particularly important, as it reflects the ensemble model’s reliability in correctly identifying true positive cases. In medical diagnosis, where false positives can lead to unnecessary treatments or invasive procedures, maintaining high precision reduces these risks and ensures that clinical interventions are directed only toward patients who truly require them. Furthermore, it achieved a high recall of 98.33% and an F1-score of 98.19%, reflecting a balanced performance between sensitivity and precision. The model also showed excellent discrimination ability, supported by its high specificity of 96.13% and elevated AUC values across test evaluations. This high specificity highlights the model’s reliability in correctly identifying non-pneumonia cases while minimizing false alarms. This is a critical factor in clinical contexts where overdiagnosis may lead to unnecessary interventions and added healthcare costs.

#### 4.3.1. Confusion Matrix Analysis

To further evaluate the model’s predictive strength on both datasets, Figure 9 presents the confusion matrix of the VGG19–MobileNetV2–SVM ensemble on CV and IT sets, along with those of the individual base models. This analysis provides a clearer view of how effectively the models distinguish between pneumonia and non-pneumonia cases and highlights the ensemble’s advantage in reducing misclassification errors.

As can be seen in (Figure 9a), the fusion model correctly identified 263 normal and 769 pneumonia samples, reducing both false positives (17 vs. 21–22) and false negatives (14 vs. 17–19) compared to the individual models. The reduction in false positives directly increases specificity, meaning fewer healthy cases were misclassified as pneumonia. The lower number of false negatives also reflects higher sensitivity.

On the IT set (Figure 9b), the fusion model further reinforced these trends. The VGG-19 + SVM misclassified 21 normal and 7 pneumonia images, while the MobileNet-V2 + SVM misclassified 16 normal and 7 pneumonia radiographs. The ensemble reduced these errors to only 7 FP and 6 FN, achieving a more balanced prediction. These improvements stem from the complementary feature representations of VGG-19 and MobileNet-V2, which capture different but relevant visual patterns. The Chi-square-based feature selection strengthened this synergy by isolating the most discriminative and strongly correlated features from both models while discarding redundant ones, enabling the SVM to achieve clearer class separation and more reliable diagnostic predictions.

#### 4.3.2. ROC-AUC and PR-AUC Analysis

**Figure 10:**
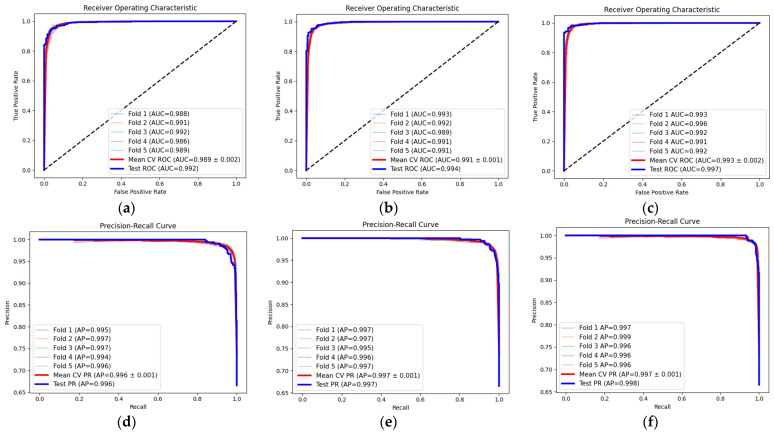
Evaluation of threshold behavior and class-imbalance performance for single models and their feature-fusion combination: Top row ROC curves illustrating the sensitivity–specificity trade-off; bottom row PR curves highlighting precision–recall performance. (**a**) VGG-19 + SVM ROC-AUC Curve; (**b**) MobileNet-V2 + SVM ROC-AUC Curve; (**c**) Feature-Fusion Ensemble + SVM ROC-AUC Curve; (**d**) VGG-19 + SVM PR-AUC Curve; (**e**) MobileNet-V2 + SVM PR-AUC Curve; (**f**) Feature-Fusion Ensemble + SVM PR-AUC Curve.

When evaluating the proposed single and fusion models on imbalanced dataset, where pneumonia samples appear more than twice as often as normal cases, it is necessary to complement threshold-based metrics with ranking metrics to obtain a fuller view of performance. In this task, it is not enough to rely on overall accuracy. The analysis must examine how well the classifier separates the two classes and how it responds when FP and FN carry different clinical costs. The ROC curve supports this by showing the model’s ability to maintain high sensitivity and low false-positive rates across thresholds. As shown in Figure 10, the VGG19 + SVM and MobileNet-V2 + SVM models achieved strong class separation, visible in the angles of their ROC curve vertices approaching 90°. The fusion model further improved this behavior, allowing the SVM to reach a mean ROC-AUC of 99.28% on the CV dataset and 99.67% on the IT set.

Although the ROC-AUC offers valuable information, it can overestimate performance in imbalanced datasets. A model may obtain a high ROC-AUC even when it struggles with the minority class because the metric is influenced by the large number of majority-class samples across thresholds. The PR-AUC becomes more informative in this context because it evaluates precision and recall for the minority class and reveals how effectively the classifier manages the imbalance. In this study, the PR-AUC results supported the trends observed in the ROC analysis, with all models reaching near-perfect values on both datasets. On the CV dataset, the VGG-19 + SVM reached a mean PR-AUC of 99.54%, MobileNetV2 + SVM reached a mean PR-AUC of 99.66%, and the fusion model increased this to 99.74%. On the test set, the scores were 99.59%, 99.71%, and 99.84% respectively. These results confirm the strong performance of all proposed models and show that the multi-model feature fusion strategy improves control over false positives and strengthens the detection of normal cases under imbalance.

#### 4.3.3. Calibration Curve

**Figure 11:**
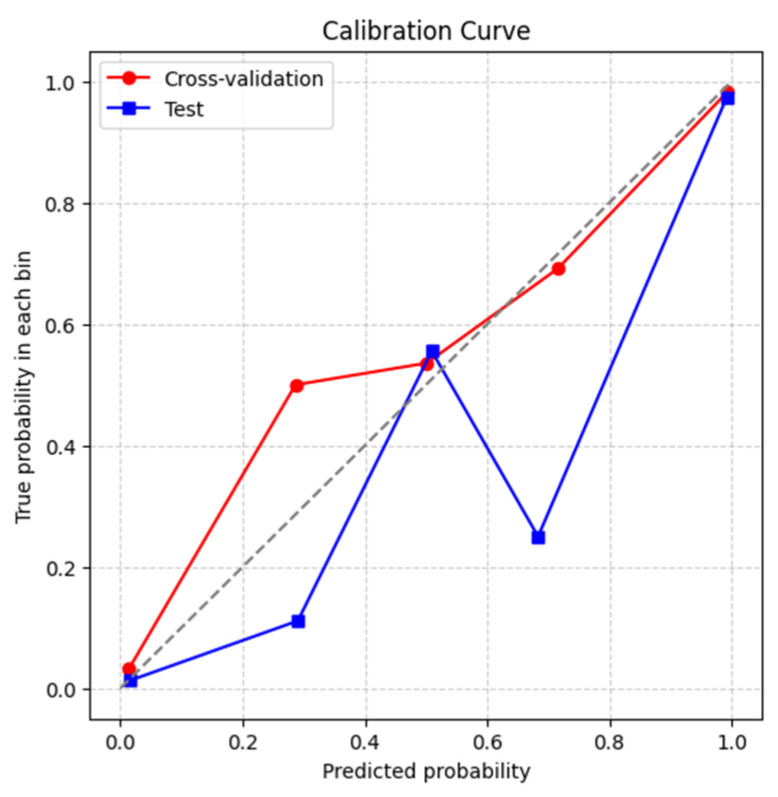
Calibration curve for the proposed feature-level fusion model with SVM classifier. The grey diagonal line represents the ideal perfectly calibrated model.

The calibration of the VGG-19 and MobileNet-V2 feature-fusion model with the SVM classifier was assessed on both cross-validation and independent test datasets, as shown in Figure 11. While probability calibration is often secondary to discrimination, it is critical in clinical decision-making because miscalibrated predictions may underestimate risk, delaying treatment, or overestimate risk, leading to unnecessary interventions.

Calibration curves, together with Brier scores, provide a comprehensive measure of predictive reliability by accounting for both discrimination and calibration.

To construct this plot, predicted probabilities were divided into five quantile bins, and for each bin the mean predicted probability was compared with the observed fraction of pneumonia cases. A perfectly calibrated model would align with the identity line. The mean Brier scores of 2.22% for CV and 2.18% for the independent test set indicate that the model generates generally well-calibrated probabilities. Minor deviations are visible in the reliability plots: the CV curve (red) remains close to the diagonal, with slight underestimation in the 0.2–0.4 range, whereas the test curve (blue) shows more noticeable overestimation around 0.7. These results suggest robust global calibration, but localized misalignments on unseen data indicate that post-calibration techniques, such as Platt scaling or isotonic regression, could further improve probability accuracy, particularly for low-to-medium risk predictions, enhancing clinical applicability.

#### 4.3.4. Ablation Study

To evaluate the impact of the Chi-square feature selection, an ablation study was performed on the proposed fusion model. Three configurations were compared: (i) selecting the top-k features using the Chi-squared test, (ii) applying PCA to reduce the feature dimensionality to k components, (iii) using the full set of features without any feature selection (no FS). Table 12 reports the classification performance for these settings.

As shown in Table 12, the Chi-2 configuration achieved the strongest performance on both datasets. It obtained the highest accuracy, F1 score, specificity, and PR-AUC, along with the lowest Brier score. PCA resulted in slightly lower performance across most metrics. The no FS configuration performed competitively on the test set and exceeded PCA in some measures, but it remained consistently below the Chi-2 setting and required many more features. Overall, the results indicate that Chi-2 selection offers the most effective balance between dimensionality reduction and predictive performance, improving both discrimination and probability reliability.

#### 4.3.5. Comparison with Other Feature Selection Methods

To assess the effectiveness of the Chi-square feature selector, we conducted a comparative analysis with several other FS approaches, including both filter and embedded methods. Among filter methods, two tests were considered. The one-way ANOVA F-test (ANOVA-F) ranks features by evaluating the variance between and within classes to identify features whose means differ significantly across groups. Mutual information Maximization (MIM) quantifies the statistical dependency between a feature and the target variable, capturing both linear and nonlinear associations, and prioritizes features that reduce uncertainty about the class label. As an embedded method, L1-regularized logistic regression (Lasso) integrates feature selection within model training by driving less informative feature coefficients to zero, effectively retaining only the most relevant predictors. Each of these methods offers distinct advantages: ANOVA-F is simple and interpretable, MIM can detect nonlinear relevance, and Lasso leverages model-specific interactions for improved generalization.

Table 13 compares the performance of Chi-square feature selection with ANOVA-F, MIM, and Lasso across the 5-fold CV and IT datasets. The results show that the Chi-square method provides the best balance between predictive accuracy and computational efficiency. ANOVA-F approach exhibited the weakest performance overall, although it was the fastest to execute. Both filter-based and embedded methods yielded strong results, confirming that the algorithms successfully captured concise and informative feature representations. Between the two filter methods, Chi-square demonstrated clear superiority in classification performance, feature compactness and execution efficiency. The Lasso method achieved a slightly higher accuracy and F1-score than Chi-square for the same number of selected features but required a significantly longer runtime, exceeding 15,000 s, making it impractical for constrained settings. On the other hand, the Chi-square test remains computationally efficient, as it does not require model training and is based only on rapid statistical calculations.

Overall, this study demonstrates the effectiveness of the Chi-square-based feature selection combined with the multi-model feature fusion strategy. Integrating selected features from multiple high-performing models consistently outperforms the use of individual models. The close agreement between cross-validation and independent test results confirms the robustness and reliability of the proposed models.

The fusion of VGG-19 and MobileNet-V2 features through the CSFEE approach offers a powerful and efficient framework for pneumonia detection, achieving a strong balance between accuracy, sensitivity, and computational efficiency, making it suitable even for resource-limited environments.

#### 4.3.6. Comparison with Other ML Classifiers

To assess the effectiveness of the SVM classifier, a comprehensive comparative analysis was conducted with 10 widely used ML classifiers, including K-Nearest Neighbors (KNN), Decision Tree (DT), Logistic Regression (LR), Nu-Support Vector Classifier (Nu-SVC), Gaussian Naive Bayes (GNB), Bagging Classifier (Bagging), Adaptive Boosting (AdaBoost), Categorical Boosting (CatBoost), Light Gradient Boosting Machin (LightGBM), and Extreme Gradient Boosting (XGBoost). All classifiers were trained using the same training data and evaluated on the same independent test set using identical evaluation metrics, including accuracy, precision, recall, F1-score and specificity, to ensure a fair comparison. Hyperparameters for each model were optimized using grid search.

Specifically, the KNN classifier was configured with n_neighbors = 175, uniform weights, brute-force search, and *p* = 2. The DT employed the Gini criterion with a maximum depth of 12, min_samples_split = 4, and min_samples_leaf = 2. LR used $L_{2}$ regularization with C = 1, tol = $3.89\times 10^{-5}$, and max_iter = 102. Nu-SVC was trained with an RBF kernel, ν = 0.35 and scale-based gamma. GNB applied a variance smoothing factor of $10^{-9}$.

**Figure 12:**
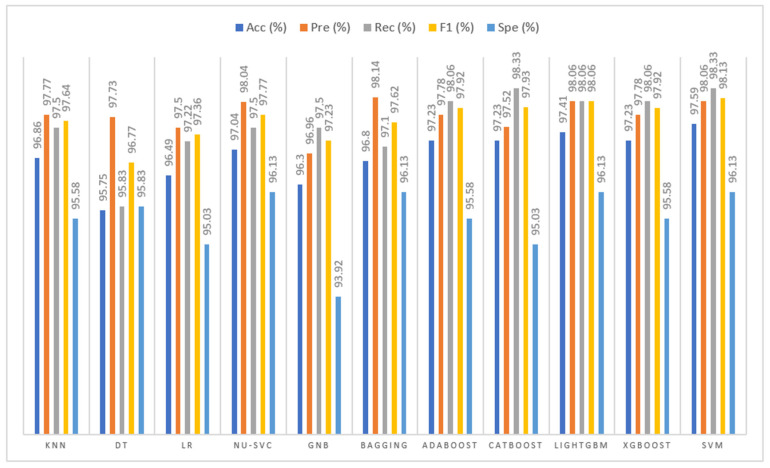
Comparison of SVM with ten widely used ML classifiers on the (IT) set.

For ensemble methods, the Bagging classifier employed 100 Decision Tree base learners with bootstrap sampling, using 80% of the training samples and 30% of the feature space for each estimator to enhance diversity and mitigate overfitting in the high-dimensional fused feature space. For boosting approaches, AdaBoost adopted decision stumps (max_depth = 1) with 100 estimators and a learning rate of 0.5 using the SAMME.R algorithm. For advanced boosting approaches, CatBoost was trained with 150 iterations, depth 6, and a learning rate of 0.05. LightGBM was configured with 150 estimators, a learning rate of 0.05, 31 leaves, and subsampling ratios of 0.8. Similarly, XGBoost employed 150 estimators, depth 4, a learning rate of 0.05, and both row and column subsampling of 0.8 and 0.3, respectively, with a binary logistic loss. As illustrated in Figure 12, despite the strong performance of these competitive baselines, the SVM classifier consistently outperforms the other models across all considered evaluation metrics, demonstrating superior generalization capability for the proposed feature space.

## 5. Discussion

This section offers a detailed evaluation of the proposed CSFFE approach, outlining its advantages and limitations relative to previous techniques presented in the literature.

### 5.1. Comparison of Different Input Image Resolutions

**Figure 13:**
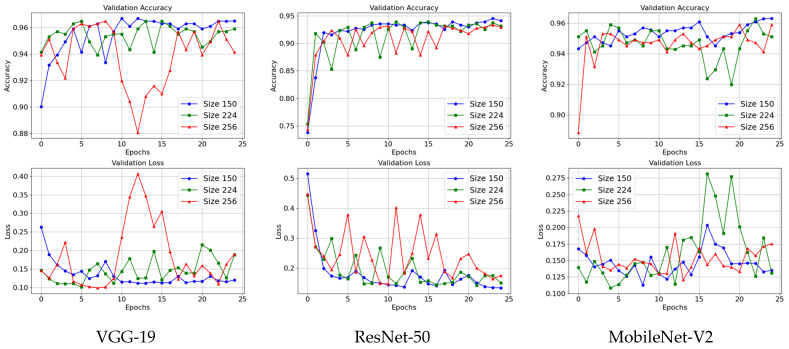
Validation accuracy and loss curves for different input sizes.

To evaluate the effect of input resolution on model performance, we trained all three models using three different input sizes: 150 × 150, 224 × 224, and 256 × 256 pixels. As illustrated in Figure 13 models trained with 150 × 150 inputs converged faster and achieved stable validation performance, suggesting that essential diagnostic features were effectively preserved despite the smaller size. Although 224 × 224 is a commonly used standard for pretrained networks and facilitates transfer learning, this resolution did not consistently improve validation accuracy and slightly increased training time and memory requirements. Inputs of 256 × 256 provided higher visual detail but did not meaningfully improve accuracy and increased computational demands, producing more fluctuations in validation metrics and slower convergence. This indicates less stable training and minimal practical benefit from using larger images. Table 14 presents a comparison of various input sizes to highlight these trade-offs.

Based on these results, 150 × 150 was selected as the optimal input size for this study, balancing efficiency with preservation of diagnostic attributes. As shown in Figure 2, the CXRs images are of high quality and sufficiently large for the clear visualization of the clinical manifestations of pneumonia.

### 5.2. Comparison with Other Research Papers

To assess the performance and effectiveness of the suggested optimal feature-level fusion ensemble model, it is crucial to compare it with state-of-the-art methods that also used the same Kermany CXR dataset for binary classification of normal and pneumonia cases. This comparison is given in Table 14. Prior works [22,23,24,26,33,37,38] reported that their ensemble-based CNN strategies outperformed current advanced algorithms.

All studies listed in Table 14 relied on the original dataset splits for training, validation, and testing. In several cases [22,23,24,26,33,37,38], training and validation sets were merged. In contrast, in [28], validation and test sets were combined. As noted in Section 3.2, these original splits contain overlapping patient IDs between training and test sets, which may cause significant data leakage. Models trained under such conditions risk learning patient-specific patterns rather than generalizable features, inflating performance metrics. In contrast, our evaluation used a rigorous protocol with 5-fold CV and a strict 90:10 patient-level split, ensuring the independent test set included only unseen patients for an unbiased assessment of model performance.

Based on the results in Table 15, the proposed CSFFE method outperformed all compared studies, achieving higher accuracy and F1 scores, with gains ranging from about 1.2 to 13.47% and 1.84 to 9.63% respectively. In addition to these improvements, the feature-level fusion strategy yielded more stable and balanced performance in pneumonia detection, demonstrating its superiority over existing cutting-edge methods applied to the same dataset.

When comparing our accuracy with that of Bhatt et al. [29] and Chouhan et al. [24], the CSFEE framework reached higher accuracy, although its recall is slightly lower. However, the number of TP detected by these two methods remains limited, as reflected in their modest precision levels of 93.28% and 80.04% respectively. It is important to highlight that the Kermany CXR dataset is heavily imbalanced, with pneumonia samples occurring more than twice as often as normal cases. In such conditions, metrics that capture the trade-off between precision and recall provide a more reliable performance assessment. The F1 score is particularly appropriate because it captures the model’s capability to handle minority cases effectively. Therefore, considering accuracy and F1 score together offers a more meaningful basis for comparison. Under this perspective, the CSFEE model clearly surpasses all reported methods, reaching an F1 score of 98.19% and an accuracy of 97.59%, confirming the robustness of our classification framework.

Recent studies proposed by Sheng et al. [27], Mabrouk et al. [22] Bodapati et al. [28] and Ayan et al. [26] demonstrated satisfactory results on the Kermany dataset, yet their performance stays below that of the CSFFE model. Mabrouk et al. [22] used a hybrid configuration that combined features extracted from MobileNet-V2, DenseNet-169, and ViT. They achieved 93.91% accuracy and a 93.43% F1 score. Their recall and specificity remained low, which affects practical utility by increasing missed cases and false alarms. Sheng et al. [27] fine-tuned three CNN backbones and merged their extracted feature maps into one representation processed by a multi-head attention module. The fused output was refined through additional attention layers and then classified by a fully connected network. Their method reached 91.67% accuracy and a 90.89% F1 score. The study also showed low recall and did not report specificity or AUC, which limits performance interpretation on an imbalanced dataset. Bodapati et al. [28] achieved stronger results than the previously discussed studies, reaching 94.84% accuracy, 95.93% F1 score, and 97.73% recall. However, their overall performance across all reported metrics did not reach the level achieved by the CSFFE model. Ayan et al. [26] achieved 95.83% accuracy and 97.76% recall using an ensemble of three pre-trained CNNs (ResNet-50, MobileNet, and Xception). While effective, their feature concatenation added complexity and redundancy, increasing the risk of overfitting on the small dataset and reducing specificity.

The channel-attention-based fusion method proposed by An et al. [23] combined residual blocks and dynamic pooling for pneumonia classification. It reached a precision of 98.38% and a specificity of 97.43%, which slightly surpassed our model on these two metrics. An et al. [23] noted a limitation in their study related to reduced recall performance due to the overuse of attention mechanisms from feature extraction to classification. As a result, the model missed several positive cases, which reduced sensitivity, as indicated by the recall of 93.84%. In contrast, our method avoids such architectural complexity. It keeps a simpler and more direct fusion strategy and reaches a recall of 98.33%, which reduces missed pneumonia cases.

To ensure a fair comparison, we also evaluated the CSFFE model on the original test split, which is prone to data leakage. Even under this setting, the proposed framework outperformed all reported state-of-the-art methods, achieving superior accuracy and F1 score while reaching a perfect recall of 100%.

To ensure a rigorous comparative assessment, we performed multiple runs using 5-fold CV while keeping the original test set fixed and varying the random shuffling of the training and validation splits. This strategy captures the natural variability of model performance. Before statistical comparison, we examined whether the performance metrics followed a normal distribution using the Kolmogorov–Smirnov test. The results showed that the normality assumption was violated. Therefore, we applied the non-parametric one-sample Wilcoxon signed-rank test with a significance level of 0.05 to evaluate whether the proposed method outperforms previous studies. As reported in Table 16, all obtained *p*-values for accuracy are below 0.05, confirming statistically significant improvements over conventional ensemble models.

### 5.3. Performance of the Proposed CSFFE Approach on Other Chest Disease Datasets

Generalization is a key requirement before any real-world implementation. The proposed CSFEE model trained on the Kermany dataset [9,10] is evaluated on external datasets of Tuberculosis [40,41], and COVID-19 & Pneumonia [42] obtained from Kaggle. The selected chi-2 features extracted from MobileNet-V2 and VGG-19 are merged and classified using the SVM classifier. The performance of the approach is assessed on each dataset using accuracy, precision, recall, f1-score, AUC, and PR-AUC. The details of the datasets are summarized in Table 17. Table 18 reports the findings for class-wise diseases in the two datasets. A comparative analysis with recent state-of-the-art approaches is presented in Table 19.

DS1: The Tuberculosis (TB) Chest X-ray Database was developed through a joint effort involving teams from Qatar University, the University of Dhaka, partners in Malaysia, and clinicians from Hamad Medical Corporation and Bangladesh. The TB CXR dataset brings together chest X-ray scans collected from several publicly available sources and contains two classes: normal cases and confirmed TB cases. The collection integrates material from the NLM Shenzhen and Montgomery datasets, the Belarus cohort, NIAID TB dataset, and selected normal scans from the RSNA pneumonia challenge set [41]. These sources provide coverage across different regions and demographic groups, offering a diverse set of chest radiographs suitable for building and evaluating TB classification systems.

DS2: The Chest X-ray (COVID-19 & Pneumonia) collection is one of the most frequently used public resources for chest disease research and was originally compiled from clinical reports and online publications to support the development of AI-based diagnostic systems. The dataset includes 6432 CXR samples grouped into three classes: 4273 Pneumonia, 576 COVID-19, and 1583 Normal, and is organized into two separate folders, namely training and testing.

The performance of the proposed CSFEE framework was evaluated on both binary and three-class classification tasks using DS1 and DS2. The results in Table 18 show that CSFEE achieves consistently high performance across different datasets and class configurations. This indicates strong robustness and effective generalization, even under class imbalance and multi-class settings.

On DS1, which focuses on binary classification between Normal and TB cases, the proposed CSFEE model shows strong and stable performance. Both classes achieve values above 98% across all evaluated metrics, with an F1-score of 99.64% for the Normal class and 98.19% for the TB class. These results demonstrate the effectiveness of combining chi-square feature selection with feature fusion from MobileNet-V2 and VGG-19. By integrating complementary representations learned at different depths and scales, CSFEE preserves discriminative patterns related to TB manifestations. This fusion strategy improves the capture of subtle radiographic signs, such as localized opacities and texture irregularities, which are essential for accurate TB identification, especially in cases where visual differences between normal and infected lungs are not pronounced.

On DS2, the classification task is more complex due to the presence of three classes and a pronounced class imbalance, particularly for COVID-19 cases. Compared to binary datasets such as DS1 and the Kermany dataset, a decline in overall accuracy is observed. This behavior is expected, as multi-class settings introduce stronger class overlap and increase the risk of confusion between visually similar patterns, especially between Normal and Pneumonia cases. Despite these challenges, the CSFEE model maintains competitive performance across all classes, achieving an overall accuracy of 95.19% and balanced precision and recall values.

The effectiveness of CSFEE on DS2 can be attributed to the integration of multi-scale features, selected via chi-square, which retain strong discriminative power. By preserving complementary patterns at different abstraction levels, the model remains sensitive to disease-specific textures and structural variations, even with the limited number of COVID-19 samples. Stable AUC (98.23%) and PR-AUC (96.01%) scores confirm its effectiveness in distinguishing classes despite the pronounced class imbalance.

The comparative results in Table 19 validate the effectiveness of CSFEE across DS1 and DS2. On DS1, the feature-level fusion of MobileNet-V2 and VGG-19 attained an average F1-score of 98.92%, markedly higher than the 94.10% achieved by the CNN-based approach [43] and the 96.00% reported for the fine-tuned VGG-16 [44].

Similarly, on DS2, the CSFEE model reached an average F1-score of 95.21%, surpassing the 92.00% obtained by the single-model VGG-16 [45]. Moreover, it outperformed the 93.89% F1-score of the ensemble framework proposed by [27], which combines DenseNet-121, ResNet-50, and VGG-19 features via multi-head attention. These findings underscore the benefits of integrating complementary multi-scale features, which maintain subtle disease-related textures often lost in standard CNNs or other ensemble methods.

### 5.4. Limitations

Despite the strong performance of the proposed approach, there are some limitations in this study. The first limitation is the exclusive use of a Chi-2 feature selection method. While efficient, it evaluates each feature separately and overlooks correlations between selected features. As a result, some redundant features may be retained in the final vector. If more filtering methods were used iteratively, they could further reduce the selected feature set and improve effectiveness. A second limitation concerns generalizability across imaging modalities, as the study relied exclusively on X-ray images. Consequently, its performance on other imaging techniques used for pulmonary disorders, such as CT scans and ultrasound, requires further validation. Finally, performance is in part influenced by class imbalance and the moderate dataset size, as no data balancing techniques were applied to better reflect real-world variability and enhance model reliability. Future research should focus on collecting larger, accurately labeled datasets from diverse sources, allowing the model to learn from authentic and varied samples.

## 6. Conclusions

Pediatric pneumonia, a serious respiratory illness, causes severe breathing difficulties. If not diagnosed in time, the risk of death can be fairly high. Recovery from pneumonia depends on a fast and accurate diagnosis. In this research, our main objective is to enhance the diagnosis of childhood pneumonia in thoracic radiographs using an effective feature selection-based multi-model feature fusion approach called CSFFE.

By applying TL and shallow FT strategies, three well-known CNN architectures were trained with hyperparameter optimization to extract features from the provided radiographs in publicly available Kermany dataset. Following the training, it was observed that the models revealed different generalization capacities and highlighted distinct salient regions from the same input radiographs. Motivated by these findings, we proposed selecting and concatenating key feature groups from each network, as this would enhance the diversity and informativeness of the feature vector used for binary classification. These new patterns vectors were then concatenated horizontally into a single matrix, exploring various combinations, and subsequently given as input to both ML and DL classifiers to determine the optimal hybrid model.

The proposed CSFFE approach proved highly effective, outperforming individual CNN models, and particularly improving the rate of negative predictions. As this research performs binary image-level classification rather than detection, our evaluation focused on complementary threshold-based metrics, including accuracy, precision, recall, F1-score, specificity, ROC-AUC, and PR-AUC. The feature-level fusion of VGG-19 and MobileNet-V2 features, combined with an SVM classifier, achieved a peak accuracy of 97.59% and an F1-score of 98.19%, with strong discrimination on ranking metrics (ROC-AUC = 99.67%) and class-wise precision–recall behavior (PR-AUC = 99.84%) on the highly imbalanced Kermany dataset.

To validate the robustness of the proposed model across both binary and three-category classification tasks, external datasets of Tuberculosis, and COVID-19 & Pneumonia were employed. DS1 had an accuracy of 99.40%, and an F1-score of 98.92%, while DS2 had an accuracy of 95.19% and an F1-score of 95.21%. Taken together, these findings emphasize CSFEE’s capacity to address challenges in chest disease classification, such as class imbalance, delicate radiographic signs, and inter-dataset variability.

Compared to related studies, CSFFE outperformed previous techniques on the same datasets in terms of accuracy, precision, and F1-score. By capturing patterns at multiple abstraction levels, the proposed hybrid ensemble improves identification of diagnostically relevant structures, providing more precise and robust classification than individual models or other ensemble approaches. Overall, these findings establish a robust, reproducible baseline for pediatric pneumonia classification and point toward dependable, AI-assisted clinical decision support.

Future work will address our model’s limitations. We plan to systematically expand the training data with diverse imaging scenarios and labeling for further enhancement. In addition, we will explore different CNN and transformer architectures, and test multi-stage feature selection to identify the most efficient hybrid architectures and key feature descriptors. Furthermore, it is essential to enhance the framework’s classification performance to improve its ability to accurately distinguish between bacterial, viral, and fungal pneumonia, which would notably assist clinicians in developing more efficient therapeutic strategies and improve patient recovery outcomes.

## Figures and Tables

**Table 1:** Comparative review of existing methods and the present work on the Kermany dataset.

Ref	Year	Architecture Type	Model Type	Class Balancing	Feature Visualization	Feature Selection	Feature Merging	Ensemble Approach	Accuracy (%)	Precision (%)	Recall (%)	F1-Score (%)
[15]	2025	Custom hybrid CNN	Hybrid Inception–ResNet CNN	No	Yes	No	Yes	None	95.35	95.35	96.41	95.35
[17]	2021	TL based CNNs	DenseNet-121	Yes/SMOTE	Yes	No	No	None	86.80	87.00	92.8	89.9
[24]	2020	Ensemble models	AlexNet, DenseNet-121, ResNet-18, Incpetion-V3, and GoogLeNet	No	Yes	No	No	Majority voting	96.39	93.28	99.62	96.35
[20]	2020	Ensemble models	Convolutions and Capsules (E4CC)	No	No	No	Yes	Convolutions and capsule feature fusion	96.36	-	-	-
[21]	2020	Ensemble models	CheXNet and VGG-19	Yes/ROS	No	Yes	Yes	Feature-level fusion	99	99	99	99
[22]	2022	Ensemble models	DenseNet-169, MobileNet-V2, and ViT	No	No	No	Yes	Feature concatenation	93.91	93.96	92.99	93.43
[26]	2022	Ensemble models	Ensemble ResNet-50, MobileNet, and Xception	Yes	Yes	No	Yes	Probabilistic voting	95.83	-	97.76	-
[28]	2022	Ensemble models	InceptionV3 + CapsNet	No	No	No	Yes	Convolutional–Capsule feature fusion	94.84	-	97.73	95.93
[29]	2023	Ensemble models	3 CNN architectures	No	No	No	No	Probability averaging	84.12	80.04	99.23	88.56
[25]	2024	Ensemble models	MobileNet, MobileNet-V2, and DenseNet-169	Yes/Undersampling	No	No	No	Soft-voting	96.31	-	-	-
[23]	2024	Ensemble models	EfficientNet-B0 and DenseNet-121	Yes/Class Weighting	Yes	No	Yes	Attention feature Fusion	95.19	98.38	93.84	96.06
[27]	2025	Ensemble models	Ensemble of DenseNet-121, ResNet-50, and VGG-19	No	No	No	Yes	Attention feature Fusion	91.67	92.19	90.00	90.89
This work	2026	Ensemble models	VGG-19 and MobileNet-V2	No	Yes	Yes	Yes	Chi-2 Selected feature fusion	97.59	98.06	98.33	98.19

**Table 2:** Data distribution of patient and image numbers across the training and testing Set.

Data Distribution	Patient Count	Images	Pneumonia	Normal
Train	2931	5315	3913	1402
Test	326	541	360	181
Total	3257	5856	4273	1583

**Table 3:** DA methods and their diagnostic relevance.

Technique	Parameters	Diagnostic Relevance
Rotation	±5° (Applied with probability 0.3)	Ensures model remains unaffected by image positioning
Shear	0.2 (Applied with probability 0.3)	Handles realistic distortions in chest anatomy
Zoom	±0.2 (Applied with probability 0.3)	Captures details at varying scales
Horizontal Flip	Applied with probability 0.5	Addresses side-specific presentation of pneumonia

**Table 4:** Impact of progressive FT layers on accuracy and computational time.

Model	Total Num of Layers	Trainable Layers	Trainable Parameters	Frozen Parameters	Acc (%)	Loss (%)	GPU Time (s)
VGG-19	26	0	4,195,329	20,024,384	94.83	22.67	551,901
		Last 5	13,634,561	10,585,152	94.90	22.59	613,923
		Last 25	24,219,713	0	94.98	22.34	693,912
		All	24,219,713	0	93.98	24.63	694,005
ResNet-50	179	0	26,215,425	23,587,712	93.60	26.58	221,833
		Last 5	27,270,145	22,532,992	95.30	17.64	228,955
		Last 25	36,205,569	13,597,568	95.58	17.39	244,227
		All	49,750,017	53,120	92.94	36.47	256,212
MobileNet-V2	158	0	16,385,025	2,257,984	93.48	21.16	205,048
		Last 5	17,105,025	1,537,984	95.12	18.38	210,816
		Last 25	17,746,945	896,064	95.19	18.23	236,847
		All	18,608,897	34,112	92.10	32.09	261,125

**Table 5:** Optimized hyperparameters with search space and selected values per models.

Classifier	Hyperparameter	Search Interval	VGG-19	ResNet-50	MobileNet-V2
FC	Learning Rate	[$10^{-5}\to 10^{-3}$] (Log scale)	$10^{-4}$	$10^{-4}$	$10^{-3}$
	Batch Size	[16 → 64] (Step of 16)	32	32	32
	Epochs	[10 → 30] (Step of 5)	25	25	25
	Dense Layers	[1 → 5] (Integer values)	2	2	3
	Units in Dense Layers	[64 → 1024] (Step factor of 2)	1024, 512	128, 256	64, 128, 256
	Dropout Rate	[0.1 → 0.5] (step of 0.1)	0.2, 0.2	0.2, 0.3	0.2, 0.3, 0.4
	Optimizer	[‘SGD’, ‘Adam’, ‘RMSprop’]	Adam	Adam	Adam
SVM	The Kernel	[‘rbf’, ‘Linear’]	Linear	Rbf	Linear
	Gamma	[‘scale’, $10^{-4}\to 1$] (log scale for numeric)	-	0.001	-
	C (Regularization)	[$10^{-4}\to 10$] (Log scale)	1.0	1.0	1.0
RF	Estimators	[100 → 500] (step of 100)	200	100	200
	Max Depth	[‘None’, 1 → 20] (Step of 1 for numeric)	None	None	None
	Min_Samples_Split	[2 → 10] (Step of 1)	2	2	2

**Table 6:** Calculation Of Chi-Square Scores for Feature Evaluation.

Positive Outcome (Pneumonia)	Negative Outcome (Normal)	Total	Total
Feature ($x_{i}$) present	$p_{1}$	$n_{1}$	$m=p_{1}+n_{1}$
Feature ($x_{i}$) absent	$p_{2}$	$n_{2}$	$t-m=p_{2}+n_{2}$
Total	$p=p_{1}+p_{2}$	$t-p=n_{1}+n_{2}$	t

**Table 7:** Comparison of the architecture of TL models used.

Model	VGG-19	ResNet-50	MobileNet-V2
Year	2014	2015	2018
Input size	224 × 224	224 × 224	224 × 224
Filters size	3 × 3	1 × 1, 3 × 3, and 7 × 7	3 × 3, 1 × 1
Depth	19	50	53
Number of FC layers	3	1	1
Number of parameters	143.7 M	25.6 M	3.5 M
Output size	7 × 7 × 512	7 × 7 × 2048	7 × 7 × 1280
Core architectural mechanism	Plain deep convolutional architecture	Deep architecture with residual skip connections	Lightweight architecture based on depthwise separable convolutions and linear bottleneck blocks
Computational profile	High	Moderate	Low

**Table 8:** Mean Performance Metrics with 95% Confidence Intervals of Single Models Evaluated Using 5-Fold Cross-Validation. The highest values are underlined.

Models	Classifier	Mean Performance Metrics of 5-Fold CV							
		Acc (%)	Pre (%)	Rec (%)	F1 (%)	Spe (%)	BS (%)	AUC (%)	PR-AUC (%)
VGG-19	FC	95.86	95.91	98.67	97.23	88.08	3.39	99.20	99.70
		[94.47, 97.28]	[93.21, 98.61]	[97.52, 99.82]	[96.37, 98.13]	[79.71, 96.45]	[2.18, 4.60]	[98.94, 99.46]	[99.58, 99.81]
	SVM	96.33	97.20	97.83	97.52	92.14	2.85	98.91	99.56
		[95.65, 97.01]	[96.38, 98.02]	[97.64, 98.02]	[97.06, 97.98]	[89.71, 94.57]	[2.56, 3.26]	[98.61, 99.21]	[99.41, 99.71]
	RF	94.26	95.22	97.09	96.14	86.37	5.17	97.89	99.09
		[93.13, 95.39]	[94.15, 96.28]	[96.21, 97.96]	[95.39, 96.89]	[83.16, 89.58]	[4.84, 5.49]	[97.59, 98.18]	[99.01, 99.17]
ResNet-50	FC	95.90	97.34	97.09	97.21	92.58	3.16	99.05	99.64
		[95.10, 96.70]	[96.67, 98.02]	[95.62, 98. 56]	[96.64, 97.77]	[90.57, 94.60]	[2.43, 3.89]	[98.69, 99.41]	[99.50, 99.78]
	SVM	96.16	96.94	97.82	97.41	91.37	3.05	98.94	99.60
		[95.33, 96.99]	[96.38, 97.49]	[97.17, 98.58]	[96.85, 97.97]	[89.79, 92.95]	[2.46, 3.63]	[98.52, 99.36]	[99.42, 99.77]
	RF	95.15	95.84	97.65	96.74	88.16	4.20	98.31	99.28
		[94.25, 96.05]	[94.78, 96.91]	[96.95, 98.35]	[96.14, 97.33]	[84.95, 91.36]	[3.79, 4.62]	[97.93, 98.69]	[99.12, 99.45]
MobileNet-V2	FC	96.16	97.33	97.47	97.40	92.51	3.31	99.03	99.63
		[95.64, 96.69]	[96.43, 98.22]	[97.03, 97.91]	[97.05, 97.74]	[89.88, 95.14]	[2.85, 3.77]	[98.93, 99.34]	[99.49, 99.77]
	SVM	96.24	97.32	97.57	97.45	92.50	2.91	99.09	99.66
		[95.76, 96.72]	[96.59, 98.05]	[96.79, 98.36]	[97.13, 97.77]	[90.30, 94.70]	[2.65, 3.05]	[98.90, 99.28]	[99.56, 99.76]
	RF	93.85	94.33	97.52	95.89	83.59	5.51	97.90	99.10
		[93.30, 94.40]	[93.26, 95.39]	[96.85, 98.19]	[95.55, 96.23]	[80.20, 86.98]	[5.25, 5.77]	[97.74, 98.06]	[98.87, 99.34]

**Table 9:** Test Performance Metrics of Single Models. The highest values are underlined.

Models	Classifier	Test Performance Metrics							
		Acc (%)	Pre (%)	Rec (%)	F1 (%)	Spe (%)	BS (%)	AUC (%)	PR-AUC (%)
VGG-19	FC	94.08	92.26	99.44	95.72	83.42	4.82	99.14	99.56
	SVM	94.82	94.38	98.05	96.18	88.39	3.62	99.17	99.59
	RF	93.53	92.87	97.77	95.26	85.08	5.04	98.72	99.36
ResNet-50	FC	95.56	95.60	97.50	96.69	91.07	3.31	99.24	99.61
	SVM	95.37	94.66	98.61	96.59	88.95	3.75	99.03	99.51
	RF	94.63	94.36	97.77	96.04	88.39	4.42	98.59	99.25
MobileNet-V2	FC	95.37	94.42	98.88	96.60	88.39	3.91	99.53	99.77
	SVM	95.74	95.66	98.05	96.84	91.16	2.93	99.42	99.71
	RF	94.63	94.13	98.04	96.05	87.84	5.64	98.57	99.30

**Table 10:** Mean Performance Metrics with 95% Confidence Intervals of Feature-Level-Fusion Ensemble Models Evaluated Using 5-Fold Cross-Validation. The highest values are underlined.

		Mean Performance Metrics of 5-Fold CV							
**Models**	***N*° of Features**	**FC Classifier**							
		**Acc (%)**	**Pre (%)**	**Rec (%)**	**F1 (%)**	**Spe (%)**	**BS (%)**	**AUC (%)**	**PR-AUC** **(%)**
VGG-19 & ResNet-50 & MobileNet-V2	Total: 2400 (800 + 800 + 800)	96.95	97.05	98.08	97.95	91.58	2.73	99.22	99.64
		[96.33, 97.57]	[96.01, 98.09]	[96.86, 99.30]	[97.53, 98.37]	[88.48, 94.68]	[2.15, 3.32]	[98.81, 99.63]	[99.32, 99.97]
VGG-19 & ResNet-50	Total: 1600 (800 + 800)	96.93	97.28	98.59	97.93	92.30	2.69	99.25	99.66
		[96.74, 97.13]	[96.91, 97.65]	[98.06, 99.13]	[97.79, 98.07]	[91.18, 93.41]	[2.58, 2.79]	[98.99, 99.51]	[99.56, 99.76]
ResNet-50 & MobileNet-V2	Total: 1000 (500 + 500)	96.52	97.45	97.83	97.64	92.86	3.21	99.27	99.60
		[95.39, 97.65]	[96.53, 98.37]	[96.46, 99.19]	[96.82, 98.46]	[90.22, 95.50]	[2.19, 4.23]	[99.18, 99.36]	[99.56, 99.64]
VGG-19 & MobileNet-V2	Total: 1600 (800 + 800)	96.73	97.44	98.13	97.78	92.80	2.87	99.16	99.65
		[96.16, 97.29]	[96.62, 98.26]	[97.50, 98.77]	[97.41, 98.16]	[90.42, 95.17]	[2.46, 3.28]	[99.05, 99.27]	[99.58, 99.72]
**Models**	***N*° of Features**	**SVM Classifier**							
		**Acc (%)**	**Pre (%)**	**Rec (%)**	**F1 (%)**	**Spe (%)**	**BS (%)**	**AUC (%)**	**PR-AUC** **(%)**
VGG-19 & ResNet-50 & MobileNet-V2	Total: 3000 (1000 + 1000 + 1000)	96.75	97.58	98.01	97.79	93.22	2.61	99.27	99.68
		[96.50, 96.99]	[97.23, 97.94]	[97.70, 98.32]	[97.63, 97.96]	[92.17, 94.27]	[2.48, 2.73]	[99.14, 99.44]	[99.54, 99.83]
VGG-19 & ResNet-50	Total: 2000 (1000 + 1000)	96.78	97.36	98.01	97.82	93.37	2.52	99.11	99.62
		[96.36, 97.21]	[97.04, 98.23]	[97.58, 98.44]	[97.53, 98.44]	[91.36, 95.11]	[2.19, 2.85]	[98.78, 99.44]	[99.45, 99.79]
ResNet-50 & MobileNet-V2	Total: 1000 (500 + 500)	96.27	97.35	97.60	97.47	92.58	2.87	99.07	99.63
		[95.47, 97.08]	[96.67, 98.03]	[96.86, 98.34]	[96.93, 98.02]	90.61, 94.55]	[2.40, 3.35]	[98.83, 99.30]	[99.52, 99.74]
VGG-19 & MobileNet-V2	Total: 2000 (1000 + 1000)	97.08	97.83	98.20	98.02	93.93	2.22	99.28	99.74
		[96.71, 97.45]	[97.11, 98.55]	[97.54, 98.86]	[97.77, 98.27]	[91.83, 96.03]	[2.07, 2.36]	[99.04, 99.53]	[99.66, 99.81]
**Models**	***N*° of Features**	**RF Classifier**							
		**Acc (%)**	**Pre (%)**	**Rec (%)**	**F1 (%)**	**Spe (%)**	**BS (%)**	**AUC (%)**	**PR-AUC** **(%)**
VGG-19 & ResNet-50 & MobileNet-V2	Total: 2700 (900 + 900+ 900)	95.90	96.41	98.08	97.24	89.80	3.72	98.81	99.51
		[95.21, 96.58]	[95.56, 97.27]	[97.20, 98.97]	[96.78, 97.70]	[87.19, 92.41]	[3.44, 4.00]	[98.61, 99.00]	[99.43, 99.59]
VGG-19 & ResNet-50	Total: 2000 (1000 + 1000)	95.86	96.37	98.08	97.22	89.66	3.73	98.69	99.44
		[95.09, 96.94]	[95.38, 97.35]	[97.63, 98.53]	[96.71, 97.72]	[86.70, 92.61]	[3.40, 4.06]	[98.43, 98.95]	[99.32, 99.56]
ResNet-50 & MobileNet-V2	Total: 2000 (1000 + 1000)	95.60	96.08	98.03	97.04	88.80	3.78	98.68	99.46
		[94.82, 96.38]	[95.00, 97.15]	[94.60, 97.52]	[96.53, 97.55]	[85.56, 92.04]	[3.51, 4.06]	[98.44, 98.91]	[99.37, 99.55]
VGG-19 & MobileNet-V2	Total: 1200 (600 + 600)	95.32	95.95	97.78	96.85	88.44	4.34	98.57	99.42
		[94.93, 95.70]	[94.99, 96.90]	[97.03, 98.52]	[96.61, 97.09]	[85.47, 91.41]	[4.13, 4.55]	[98.47, 98.67]	[99.33, 99.51]

**Table 11:** Test Performance Metrics of Feature-Level-Fusion Ensemble Models. The highest values are underlined.

		Test Performance Metrics							
**Models**	***N*° of Features**	**FC Classifier**							
		**Acc (%)**	**Pre (%)**	**Rec (%)**	**F1 (%)**	**Spe (%)**	**BS (%)**	**AUC (%)**	**PR-AUC** **(%)**
VGG-19 & ResNet-50 & MobileNet-V2	Total: 2400 (800 + 800 + 800)	97.04	96.48	99.16	97.80	92.81	2.65	99.56	99.81
VGG-19 & ResNet-50	Total: 1600 (800 +800)	96.48	96.96	97.97	97.37	93.92	2.61	99.50	99.76
ResNet-50 & MobileNet-V2	Total: 1000 (500 + 500)	97.21	97.49	98.33	97.86	95.08	2.50	99.64	99.80
VGG-19 & MobileNet-V2	Total: 1600 (800 + 800)	97.04	97.25	98.33	97.79	94.47	2.61	99.65	99.83
**Models**	***N*° of Features**	**SVM Classifier**							
		**Acc (%)**	**Pre (%)**	**Rec (%)**	**F1 (%)**	**Spe (%)**	**BS (%)**	**AUC (%)**	**PR-AUC** **(%)**
VGG-19 & ResNet-50 & MobileNet-V2	Total: 3000 (1000 + 1000 + 000)	97.22	97.52	98.33	97.92	95.13	2.40	99.52	99.76
VGG-19 & ResNet-50	Total: 2000 (1000 + 1000)	96.11	95.68	97.61	97.12	91.16	2.74	99.51	99.75
ResNet-50 & MobileNet-V2	Total: 1000 (500 + 500)	97.50	97.52	97.87	98.06	94.47	2.23	99.65	99.83
VGG-19 & MobileNet-V2	Total: 2000 (1000 + 1000)	97.59	98.06	98.33	98.19	96.13	2.18	99.67	99.84
**Models**	***N*° of Features**	**RF Classifier**							
		**Acc (%)**	**Pre (%)**	**Rec (%)**	**F1 (%)**	**Spe (%)**	**BS (%)**	**AUC (%)**	**PR-AUC** **(%)**
VGG-19 & ResNet-50 & MobileNet-V2	Total: 2700 (900 + 900 + 900)	95.93	95.43	97.61	96.99	90.60	3.79	99.29	99.65
VGG-19 & ResNet-50	Total: 2000 (1000 + 1000)	95.37	94.66	97.53	96.59	88.95	3.97	99.11	99.54
ResNet-50 & MobileNet-V2	Total: 2000 (1000 + 1000)	95.56	94.91	97.33	96.73	89.50	3.88	99.17	99.57
VGG-19 & MobileNet-V2	Total: 1200 (600 + 600)	95.74	95.41	96.30	96.85	90.60	4.28	99.10	99.57

**Table 12:** Ablation Study of Dimensionality Reduction Methods for the Proposed Fusion Model. The highest values are underlined.

		**Mean Performance Metrics of 5-Fold CV**							
**FS Method**	***N*° of Features**	**Acc (%)**	**Pre (%)**	**Rec (%)**	**F1 (%)**	**Spe (%)**	**BS (%)**	**AUC (%)**	**PR-AUC** **(%)**
Chi-2	Total: 2000 (1000 + 1000)	97.08	97.83	98.20	98.02	93.93	2.22	99.28	99.74
		[96.71, 97.45]	[97.11, 98.55]	[97.54, 98.86]	[97.77, 98.27]	[91.83, 96.03]	[2.07, 2.36]	[99.04, 99.53]	[99.66, 99.81]
PCA	Total: 2000 (1000 + 1000)	96.71	97.59	97.96	97.77	93.22	2.70	99.17	99.69
		[96.41, 97.01]	[96.95, 98.22]	[97.51, 98.40]	[97.57, 97.96]	[91.36, 95.09]	[2.49, 2.91]	[98.94, 99.40]	[99.69, 99.80]
No FS	Total: 40,192 (8192 + 32,000)	96.29	97.41	97.57	97.49	92.72	2.80	99.01	99.56
		[95.55, 97.04]	[96.25, 98.57]	[97.02, 98.12]	[97.00, 97.98]	[89.33, 96.12]	[2.45, 3.15]	[98.83, 99.20]	[99.37, 99.74]
**FS Method**	***N*° of Features**	**Test Performance Metrics**							
		**Acc (%)**	**Pre (%)**	**Rec (%)**	**F1 (%)**	**Spe (%)**	**BS (%)**	**AUC (%)**	**PR-AUC** **(%)**
Chi-2	Total: 2000 (1000 + 1000)	97.59	98.06	98.33	98.19	96.13	2.18	99.67	99.84
PCA	Total: 2000 (1000 + 1000)	96.48	96.20	98.61	97.39	92.26	2.75	99.37	99.69
No FS	Total: 40,192 (8192 + 32,000)	96.11	96.43	97.77	97.10	92.81	2.59	99.52	99.77

**Table 13:** Comparative evaluation of feature selection methods in terms of performance and efficiency on 5-fold CV and test datasets.

		**Mean Performance Metrics of 5-Fold CV**						
**FS Method**	***N*° of Features**	**Acc (%)**	**F1 (%)**	**Spe (%)**	**BS (%)**	**AUC (%)**	**PR-AUC** **(%)**	**Time (s)**
Filter: Chi-2	Total: 2000 (1000 + 1000)	97.08	98.02	93.93	2.22	99.28	99.74	108.74
		[96.71, 97.45]	[97.77, 98.27]	[91.83, 96.03]	[2.07, 2.36]	[99.04, 99.53]	[99.66, 99.81]	
Filter: ANOVA-F	Total: 1800 (900 + 900)	95.39	96.88	90.44	3.51	98.74	99.51	58.55
		[95.00, 95.78]	[96.62, 97.14]	[89.01, 91.87]	[3.11, 3.91]	[98.46, 99.01]	[99.38, 99.64]	
Filter: MIM	Total: 2200 (1100 + 1100)	96.80	97.83	93.30	2.55	99.12	99.61	483.41
		[96.47, 97.13]	[97.61, 98.06]	[91.54, 95.05]	[2.36, 2.75]	[98.85, 99.39]	[99.43, 99.80]	
Embedded: Lasso	Total: 2000 (1000 + 1000)	97.28	98.15	93.97	2.17	99.41	99.78	1,551,560
		[97.12, 97.44]	[98.04, 98.26]	[92.80, 95.14]	[1.97, 2.37]	[99.27, 99.54]	[96.72, 99.84]	
**FS Method**	***N*° of Features**	**Test Performance Metrics**						
		**Acc (%)**	**F1 (%)**	**Spe (%)**	**BS (%)**	**AUC (%)**	**PR-AUC** **(%)**	**Time (s)**
Filter: Chi-2	Total: 2000 (1000 + 1000)	97.59	98.19	96.13	2.18	99.67	99.84	24.15
Filter: ANOVA-F	Total: 1800 (900 + 900)	94.82	96.17	88.95	3.97	98.77	99.37	17.18
Filter: MIM	Total: 2200 (1100 + 1100)	97.04	97.79	94.47	2.19	99.65	99.83	30.23
Embedded: Lasso	Total: 2000 (1000 + 1000)	97.78	98.34	96.11	2.17	99.65	99.82	60.38

**Table 14:** Comparison of different input image resolutions: balancing diagnostic feature retention and computational demand.

Input Size	Preservation of Diagnostic Features	Processing Cost	Prevalence in Literature	Training Speed	Performance in Disease Classification
150 × 150	Relevant patterns retained	Minimal	Rarely adopted	Rapid	Reliable and stableperformance
224 × 224	Additional structural detailscaptured	Intermediate	Widely used	Moderate	Comparable accuracy with stable outcomes
256 × 256	Advanced details available with limited added diagnostic value	Substantial	Occasionallyreported	Slower	No appreciableimprovement; training stability reduced

**Table 15:** Performance comparison of the proposed feature-fusion ensemble framework with existing CNN ensemble methods on the Kermany dataset.

Ref	Year	Test Size	Classification Type	Techniques	Acc (%)	Pre (%)	Rec (%)	F1 (%)	Spe (%)	AUC (%)
[24]	2020	624	BN	Majority voting	96.39	93.28	99.62	96.35	-	99.34
[22]	2022	624	BN	Feature fusion	93.91	93.96	92.99	93.43	89.32 *	-
[28]	2022	640	BN	InceptionV3 + CapsNet	94.84	-	97.73	95.93	-	93.9
[26]	2022	624	BN	Ensemble of ResNet-50, MobileNet, and Xception	95.83	-	97.76	-	92.73	95.21
[29]	2023	624	BN	Ensemble of 3 CNN architectures	84.12	80.04	99.23	88.56	-	-
[23]	2024	624	BN	Channel attention-based feature fusion	95.19	98.38	93.84	96.06	97.43	95.64
[27]	2025	624	BN	Feature Fusion based on multi-head attention mechanism	91.67	92.19	90.00	90.89	-	-
Proposed CSFFE	2025	541	BN	Selected feature fusion	97.59	98.06	98.33	98.19	96.13	99.67
		624	BN	Selected feature fusion	97.59	96.29	1.00	98.11	93.58	99.92

* Computed based on the confusion matrix presented in the article.

**Table 16:** Statistical comparison of classification performance using the Wilcoxon Signed-Rank Test.

Study (S)	S1 [24]	S2 [22]	S3 [28]	S4 [26]	S5 [29]	S6 [23]	S7 [27]
*p*-value	$3.15\times 10^{-6}$	$8.62\times 10^{-7}$	$8.62\times 10^{-7}$	$1.17\times 10^{-6}$	$8.62\times 10^{-7}$	$8.62\times 10^{-7}$	$8.62\times 10^{-7}$

**Table 17:** Details of pneumonia-related CXR datasets used in this study.

Dataset	Number of CXRs	Classes	Disease	Link
DS1: TB CXR	3500 Normal, 700 Tuberculosis	2	Tuberculosis	https://www.kaggle.com/datasets/tawsifurrahman/tuberculosis-tb-chest-xray-dataset/data (accessed on 9 February 2026) [39]
DS2: COVID-19 & Pneumonia	576 COVID-19, 1583 Normal, 4273 Pneumonia	3	COVID-19 & Pneumonia	https://www.kaggle.com/datasets/prashant268/chest-xray-covid19-pneumonia/data (accessed on 9 February 2026) [42]

**Table 18:** Class-wise performance of the VGG-19 and MobileNet-V2 feature fusion using an SVM classifier across different pneumonia datasets.

Dataset	Data Used	Size	Train/Test Split	Support	Classes	Acc (%)	Pre (%)	Rec (%)	F1 (%)	AUC (%)	PR-AUC (%)
DS1	100%	4200	[80:20]	700	Normal	99.40	99.43	99.86	99.64	-	-
				140	Tuberculosis		99.27	97.14	98.19	-	-
DS2	100%	6432	[80:20]	116	COVID-19	95.19	99.12	96.55	97.82	98.23	96.01
				317	Normal		89.39	93.06	91.19	94.73	84.9
				855	Pneumonia		96.92	95.79	96.35	94.89	95.64

**Table 19:** Comparison of CSFFE with cutting-edge methods on binary and three-class datasets.

Dataset	Ref	Year	Techniques	Data Used	Acc (%)	Pre (%)	Rec (%)	F1 (%)	AUC (%)	PR-AUC (%)
DS1	[43]	2024	CNN	28%: 563 Normal, 633 Tuberculosis	94.00	93.40	96.85	94.10	-	-
	[44]	2024	VGG-16	100%: 3500 Normal, 700 Tuberculosis	98.00	98.00	94.00	96.00	-	-
	Proposed	2026	CSFEE	100%: 3500 Normal, 700 Tuberculosis	99.40	99.35	98.50	98.92	99.86	99.48
DS2	[45]	2021	VGG-16	100%: 576 COVID-19, 1583 Normal, 4273 Pneumonia	92.00	92.00	92.00	92.00	-	-
	[27]	2025	Ensemble	100%: 576 COVID-19, 1583 Normal, 4273 Pneumonia	93.79	92.43	95.99	93.89	-	-
	Proposed	2026	CSFEE	100%: 576 COVID-19, 1583 Normal, 4273 Pneumonia	95.19	95.27	95.19	95.21	95.95	92.18

## Data Availability

The data presented in this study are openly available in Kaggle at the following links: Chest X-ray Pneumonia dataset: https://www.kaggle.com/datasets/paultimothymooney/chest-xray-pneumonia, accessed on 28 August 2025. The Tuberculosis (TB) Chest X-ray: https://www.kaggle.com/datasets/tawsifurrahman/tuberculosis-tb-chest-xray-dataset/data, accessed on 10 December 2025. The Chest X-ray (COVID-19 & Pneumonia): https://www.kaggle.com/datasets/prashant268/chest-xray-covid19-pneumonia/data, accessed on 8 December 2025. Code Availability: The Python code used in this study, including preprocessing, model training, and evaluation scripts, will be made available by the first author upon reasonable request. License Information: The original dataset is publicly available on Mendeley Data at the following links: https://data.mendeley.com/datasets/rscbjbr9sj/2, (accessed on 21 August 2025) and is distributed under the Creative Commons Attribution 4.0 International License (CC BY 4.0).

## Abbreviations

The following abbreviations are used in this manuscript:

CXR	Chest radiography
CNN	Convolutional neural networks
Chi-2	Chi-square test
AI	Artificial intelligence
DL	Deep learning
TL	Transfer learning
FN	Fine-tuning
ML	Machine Learning
FF	Feature Fusion
FS	Feature Selection
CSFFE	Chi-square selected feature fusion ensemble
CV	Cross Validation
IT	Independent test set
Grad-CAM	Gradient-weighted Class Activation Mapping
XAI	Explainable AI
DA	Data augmentation
